# Genome-Wide and Follow-Up Studies Identify *CEP68* Gene Variants Associated with Risk of Aspirin-Intolerant Asthma

**DOI:** 10.1371/journal.pone.0013818

**Published:** 2010-11-03

**Authors:** Jeong-Hyun Kim, Byung-Lae Park, Hyun Sub Cheong, Joon Seol Bae, Jong Sook Park, An Soo Jang, Soo-Taek Uh, Jae-Sung Choi, Yong-Hoon Kim, Mi-Kyeong Kim, Inseon S. Choi, Sang Heon Cho, Byoung Whui Choi, Choon-Sik Park, Hyoung Doo Shin

**Affiliations:** 1 Department of Life Science, Sogang University, Seoul, Republic of Korea; 2 Department of Genetic Epidemiology, SNP Genetics Incorporation, Seoul, Republic of Korea; 3 Division of Allergy and Respiratory Medicine, Soonchunhyang University Bucheon Hospital, Bucheon, Republic of Korea; 4 Division of Allergy and Respiratory Medicine, Soonchunhyang University Cheonan Hospital, Cheonan, Republic of Korea; 5 Division of Internal Medicine, Chungbuk National University, Cheongju, Republic of Korea; 6 Department of Allergy, Chonnam National University, Gwangju, Republic of Korea; 7 Department of Internal Medicine, Seoul National University, Seoul, Republic of Korea; 8 Department of Internal Medicine, Chung-Ang University Yongsan Hospital, Seoul, Republic of Korea; GSF National Research Center for Environment and Health, Germany

## Abstract

Aspirin-intolerant asthma (AIA) is a rare condition that is characterized by the development of bronchoconstriction in asthmatic patients after ingestion of non-steroidal anti-inflammatory drugs including aspirin. However, the underlying mechanisms of AIA occurrence are still not fully understood. To identify the genetic variations associated with aspirin intolerance in asthmatics, the first stage of genome-wide association study with 109,365 single nucleotide polymorphisms (SNPs) was undertaken in a Korean AIA (n = 80) cohort and aspirin-tolerant asthma (ATA, n = 100) subjects as controls. For the second stage of follow-up study, 150 common SNPs from 11 candidate genes were genotyped in 163 AIA patients including intermediate AIA (AIA-I) subjects and 429 ATA controls. Among 11 candidate genes, multivariate logistic analyses showed that SNPs of *CEP68* gene showed the most significant association with aspirin intolerance (*P* values of co-dominant for *CEP68*, 6.0×10^−5^ to 4.0×10^−5^). All seven SNPs of the *CEP68* gene showed linkage disequilibrium (LD), and the haplotype of *CEP68_ht4* (T-G-A-A-A-C-G) showed a highly significant association with aspirin intolerance (OR  = 2.63; 95% CI  = 1.64–4.21; *P* = 6.0×10^−5^). Moreover, the nonsynonymous *CEP68* rs7572857G>A variant that replaces glycine with serine showed a higher decline of forced expiratory volume in 1s (FEV_1_) by aspirin provocation than other variants (*P* = 3.0×10^−5^). Our findings imply that *CEP68* could be a susceptible gene for aspirin intolerance in asthmatics, suggesting that the nonsynonymous Gly74Ser could affect the polarity of the protein structure.

## Introduction

Aspirin, also known as acetylsalicylic acid, has been used as an analgesic to relieve pain and fever, as well as an anti-inflammatory medication. Up to 20% of asthmatics are sensitive to aspirin and other non-steroidal anti-inflammatory drugs (NSAIDs). Aspirin-intolerant asthma (AIA), as a unique clinical syndrome with acute bronchospasm after ingestion of aspirin or other NSAIDs, was first described in 1922 [1], [2]. AIA is characterized by the triad of aspirin hypersensitivity, bronchial asthma, and chronic rhinosinusitis with nasal polyposis [3], [4]. AIA progression from the upper to the lower respiratory tract is accompanied by persistent asthmatic symptoms with intense eosinophilic infiltration into the upper and lower airways. AIA generally begins at about 30 years of age and occurs more frequently in women [2], [5].

Recent studies have shown that aspirin hypersensitivity is likely to be related with over-production of pro-inflammatory cysteinyl leukotrienes (CysLTs) such as LTC4, LTD4, and LTE4 [2]. CysLTs are generated from arachidonic acid via the leukotriene synthetic pathway or the 5-lipoxygenase pathway. Aspirin blocks the cyclooxygenase pathway that converts arachidonic acid into prostaglandins, and eventually diverts arachidonic acid metabolites to the 5-lipoxygenase pathway [6], [7]. Therefore, CysLT receptors and arachidonic acid cascade-related enzymes/proteins have been considered as main targets for aspirin intolerance in asthmatics. CysLT receptors are selectively antagonized by several leukotriene modifiers, such as montelukast, pranlukast, and zafirlukast [8], [9]; however, clinical studies have demonstrated that the responses to these drugs is incomplete [10], [11], suggesting the presence of an alternative pathway leading to AIA. Previous genetic studies have revealed significant associations between AIA and the polymorphisms of cysteinyl leukotriene receptor 1 [12], cysteinyl leukotriene receptor 2 [13], thromboxane A2 receptor [14], and prostaglandin E2 receptor subtype 2 [15]. The genetic polymorphisms of leukotriene C4 synthase (*LTC4S*) [16], [17], [18] and arachidonate 5-lipoxygenase (*ALOX5*) [19], [20] have been reported to be positively or negatively associated with AIA depending on populations and polymorphic loci. In addition to the genes on the arachidonate pathway, genetic variants on *TNF* [21], *HLA* [22], [23], [24], *TBX21* [25], *ACE* [26], *IL-10/TGF* [27], and *NLRP3* [28] genes in the pathways of immune response and inflammation are also associated with aspirin hypersensitivity in asthmatics. These data suggest that genetic variants of genes on other pathways may be more relevant to development of aspirin hypersensitivity in asthmatics than was previously thought.

Genome-wide association studies (GWAS) have recently emerged as a technology in predicting genetic variations across the genome that are associated with human diseases and clinical response to drug treatment [29], [30]. Recently, GWAS for asthma and related phenotypes have reported several susceptible genes including *ORMDL3*, *PDE4D* and *IL1RL1* [31], [32], [33]. In the case of AIA, most of the recent genetic association analyses have been investigated through candidate gene approaches. Therefore, genome-wide association analysis and follow-up study of aspirin intolerance in asthmatics were performed in order to identify novel and comprehensive etiology of AIA.

## Results

### Characteristics of the Study Subjects

The clinical characteristics and comparisons of AIA, intermediate AIA (AIA-I), and ATA groups are summarized in Table 1. First of all, an aspirin-induced decline in FEV_1_ of −15% to 68% was observed in all study subjects. In both of genome-wide and follow-up studies, the % declines of FEV_1_ by aspirin provocation in AIA and AIA-I patients were significantly increased compared to those of ATA controls (*P*<0.0001), indicating that this study could reflect an association between genetic polymorphisms and aspirin hypersensitivity in asthmatics. In subjects of 2nd stage, the values of predicted FEV_1_ % and PC20 methacholine were significantly lower in AIA patients than those of ATA controls, whereas the total IgE level was higher in cases than in controls (*P*<0.01). A significant decrease of body mass index (BMI) in aspirin-intolerant asthmatics was also observed. In addition, the mean age of first medical examination was significantly lower in AIA than in the ATA group.

**Table 1:** Clinical profiles of aspirin-intolerant and aspirin-tolerant asthmatics.

Clinical profile	First stage of genome-wide study		Second stage of follow-up study				
	AIA	ATA	Asthmatics(all subjects)	AIA		ATA	
				AIA	AIA-I		
Number of subjects (n)	80	100	592	102	61	429	
Age of onset [year, mean (range)]	45.06 (17.22–71.56)	45.56 (15.41–77.13)	46.15 (15.40–77.88)	42.76 (18.66–72.73) **	43.73 (17.22–71.29)	47.30 (15.40–77.88)	
Sex (n, male/female)	27/53	27/73	206/386	37/65	22/39	147/282	
Current Smoker (%)	20.00	26.00	27.70	20.59	22.95	30.07	
Height [cm, mean (range)]	161.82±9.90	159.75±8.45	160.78±8.63	161.70±9.91	161.75±7.95	160.42±8.39	
Weight (kg)	61.74±10.16	62.41±10.32	62.81±10.84	61.64±10.39	60.61±10.42	63.40±10.97	
Body mass index (kg/m2)	23.57±3.20	24.44±3.39	24.24±3.39	23.56±3.37**	23.09±3.06**	24.58±3.39	
% decline of FEV1 by aspirin provocation	25.84±14.00***	0.95±2.77	9.27±13.24	33.59±13.42***	10.07±6.74***	3.54±4.85	
Blood eosinophil (%)	6.87±5.79*	4.89±4.16	6.01±5.73	6.65±5.78	4.80±3.89	6.03±5.92	
FEV1 (% predicted)	82.53±21.90	82.09±20.69	90.54±16.97	85.10±16.41**	91.73±17.12	91.66±16.87	
PC20 methacholine (mg/ml)	3.88±6.80	2.83±3.59	6.43±8.67	4.26±7.62**	6.20±8.06	6.91±8.90	
Log[Total IgE (IU/ml)]	2.21±0.52	2.14±0.62	2.16±0.63	2.26±0.58	2.15±0.45	2.13±0.66	
Positive rate of skin test (%)	58.75	63.00	56.42	48.04	60.66	57.81	

Each clinical profile of AIA and AIA-I was compared to ATA controls, respectively.

*P<0.05;

**P<0.01;

***P<0.0001.

AIA, aspirin-intolerant asthma; AIA-I, intermediate aspirin-intolerant asthma; ATA, aspirin-tolerant asthma.

### Genome-Wide Association Analyses

A total of 109,365 SNPs genotype assays were tested on the DNA samples of 80 AIA and 100 ATA subjects using the Illumina's Human-1 Genotyping BeadChip. The overall call rate of individual samples was over 98.0% after passing the genotype quality threshold, and a total of 4,515 SNPs (4.1% of 109,365 SNPs) failed to provide accurate genotype results. In further quality control analysis of the remaining 104,850 SNPs, monomorphic, X-chromosomal and Hardy Weinberg Equilibrium-departed (*P*<0.001) SNPs were additionally omitted; the final remaining 96,984 SNPs (88.7%) with an the average call rate of 99.9% were selected for analysis.

From results of the allelic association tests for each SNP in the co-dominant model, the 11 SNPs in the gene region that had the most significant association signals were screened for the second stage of follow-up study (Table 2).

**Table 2:** Top 11 Genetic variants within gene regions with lowest *P* value for risk of aspirin intolerance.

SNP ID	Gene	Chr. locus	Variation	Position	Function	MAF		*P* value*
						AIA (n = 80)	ATA (n = 100)	
rs1053744	*SBF1*	22q13.33	C>T	Exon39	SET binding factor 1	0.500	0.365	3.0×10^−7^
rs828616	*DCBLD2*	3q12.1	A>G	Exon 6	Discoidin, CUB and LCCL domain containing 2	0.195	0.365	2.0×10^−6^
rs3213729	*WDR21A*	14q24.3	C>G	Promoter	DDB1 and CUL4 associated factor 4	0.230	0.100	1.0×10^−5^
rs1932523	*FILIP1*	6q14.1	T>C	Intron1	Filamin A interacting protein 1	0.465	0.290	3.0×10^−5^
rs4867084	*PDZK3*	5p13.3	G>A	Intron1	PDZ domain containing 2	0.200	0.365	4.0×10^−5^
rs11060167	*LRRC43*	12q24.31	C>A	Exon8	Leucine rich repeat containing 43	0.225	0.380	4.0×10^−5^
rs6498124	*CIITA*	16p13.13	G>T	Intron6	Class II, major histocompatibility complex, transactivator	0.350	0.535	4.0×10^−5^
rs2564978	*DAF*	1q32.2	T>C	Promoter	CD55 molecule, decay accelerating factor for complement	0.535	0.390	5.0×10^−5^
rs2295017	*ENPP5*	6p12.3	A>T	3′UTR	Ectonucleotide pyrophosphatase/phosphodiesterase 5	0.090	0.215	5.0×10^−5^
rs2252867	*CEP68*	2p14	A>G	Intron1	Centrosomal protein 68 kDa	0.415	0.250	7.0×10^−5^
rs4245976	*C6*	5p13	C>T	Intron6	Complement component 6	0.250	0.156	7.0×10^−5^

MAF, minor allele frequency; Chr., chromosome.

*P values of co-dominant model. UTR; untranslated region.

### Follow-up Study of Second Stage

For the follow-up study, the 11 genetic variants “within gene regions” with the lowest *P* value (*P*<0.0001) for risk of aspirin intolerance were selected. Then, 150 common SNPs in 11 candidate genes with minor allele frequency of over 0.05 based on Asian population were selected from the International HapMap Project (http://hapmap.ncbi.nlm.nih.gov/) and genotyped in 163 AIA subjects including AIA-I group, and 429 ATA subjects. In order to include the maximum number of patients with aspirin hypersensitivity, 80 AIA and 100 ATA subjects from the first stage were included in the second stage due to the rareness of the AIA condition. The case/control associations between genotype and aspirin intolerance in asthmatics were analyzed using multivariate logistic analyses adjusted for initial diagnosed age, sex, smoking status, atopy, and BMI. Polymorphisms of the CEP68 gene showed the most significant association with AIA compared to ATA controls in co-dominant model (Table 3, P = 6.0×10^−5^ to 4.0×10^−5^). Furthermore, a nonsynonymous SNP (rs7572857G>A) of *CEP68* gene that replaces glycine with serine revealed to have the lowest *P* value among seven SNPs of the gene (OR  = 2.63; 95% CI  = 1.64–4.21; *P* = 6.0×10^−5^ for co-dominant model). This rs7572857G>A was also statistically significant in the dominant model (*P* = 0.0005), but not in recessive model (Table S1). In further association analysis excluding the GWAS subjects in the second set, the statistically significant association still remained although reduced significances were observed (Table S2).

**Table 3:** Logistic analyses of SNPs in the *CEP68* gene between AIA and ATA.

	Position	Amino acid change	AIA (n = 102) vs. ATA (n = 429)					[AIA+AIA-I] (n = 163) vs. ATA (n = 429)				
			Frequency^a^		Co-dominant model			Frequency^a^		Co-dominant model		
			AIA	ATA	OR (95% CI)	*P* *	*P^corr^* **	[AIA+AIA-I]	ATA	OR (95% CI)	*P* *	*P^corr^* **
**SNP**												
rs2302647 C>T	Promoter		0.456	0.309	1.82 (1.32–2.50)	**0.0002**	**0.001**	0.399	0.309	1.46(1.12–1.91)	**0.006**	**0.02**
rs2252867 A>G	Intron1		0.461	0.319	1.78 (1.30–2.45)	**0.0004**	**0.002**	0.414	0.319	1.50(1.15–1.96)	**0.003**	**0.01**
rs12611491 A>G	Exon2	Arg27Gly	0.284	0.241	1.25 (0.88–1.79)	0.22	-	0.285	0.241	1.28(0.95–1.74)	0.1	-
rs7572857 G>A	Exon2	Gly74Ser	0.176	0.077	2.63 (1.64–4.21)	**6.0×10^−5^**	**0.0003**	0.126	0.077	1.71(1.11–2.62)	**0.02**	-
rs2723087 T>A	Intron2		0.461	0.319	1.78 (1.30–2.45)	**0.0004**	**0.002**	0.414	0.319	1.50(1.15–1.96)	**0.003**	**0.01**
rs6741255 T>C	Intron5		0.466	0.317	1.87 (1.35–2.57)	**0.0001**	**0.0005**	0.417	0.317	1.55(1.18–2.03)	**0.002**	**0.007**
rs10496123 G>A	Intron5		0.289	0.339	0.81 (0.58–1.14)	0.23	-	0.304	0.339	0.85(0.64–1.13)	0.27	-
**Haplotype**												
* CEP68_ht1*			0.240	0.340	0.60(0.42–0.86)	**0.006**		0.276	0.340	0.73(0.54–0.97)	**0.03**	
* CEP68_ht2*			0.289	0.331	0.84(0.60–1.17)	0.3		0.304	0.331	0.88(0.66–1.17)	0.38	
* CEP68_ht3*			0.275	0.218	1.34(0.93–1.93)	0.12		0.267	0.218	1.32(0.97–1.79)	0.08	
* CEP68_ht4*			0.176	0.077	2.63(1.64–4.21)	**6.0×10^−5^**		0.126	0.077	1.71(1.11–2.62)	**0.02**	

a The frequency of SNP indicates the minor allele frequency.

*P values represent the co-dominant model, which includes the additive model, adjusted for age at initial diagnosis, sex, smoking status, atopy, and body mass index.

**P^corr^ values after multiple testing correction.

AIA, aspirin-intolerant asthma; AIA-I, intermediate aspirin-intolerant asthma; ATA, aspirin-tolerant asthma; OR, odds ratio; CI, confidence interval.

**Figure 1:**
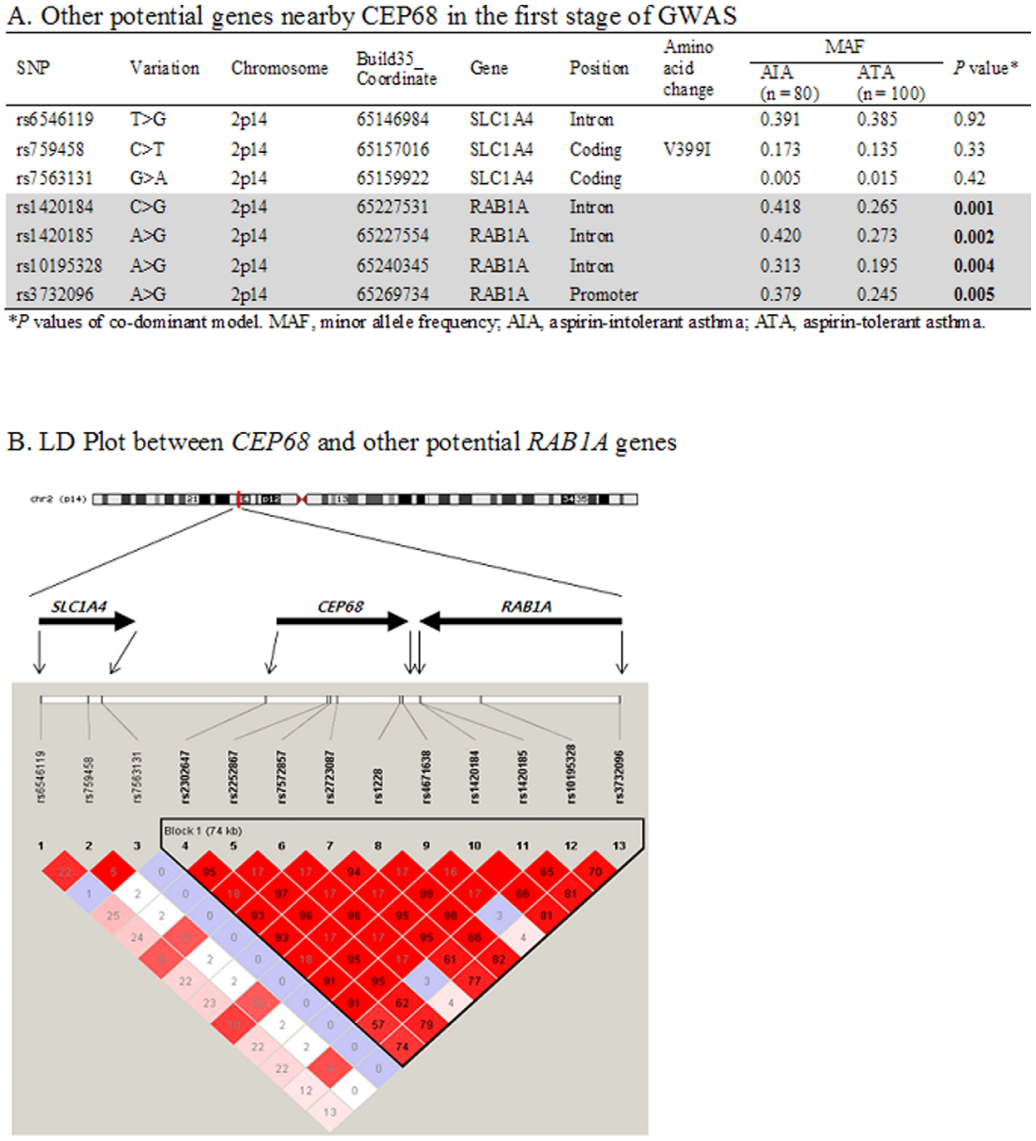
Other potential candidate gene nearby *CEP68*. (A) Associations of other potential genes nearby *CEP68*. A significant association of SNPs in *RAB1A* is found from the result of this GWAS (*P*<0.01). (B) LD Plot between *CEP68* and *RAB1A.* LD coefficient (D') among SNPs of *SLC1A4*, *RAB1A* and *CEP68* in Korean population.

This study also observed modest associations of other genes with AIA. In particular, the intronic rs4867084G>A of the *PDZK3* gene (*P* = 0.006 for co-dominant model) and rs11060333C>T of the *LRRC43* gene (*P* = 0.009 for co-dominant model) showed significant associations with AIA (Table S3). In addition, several intergenic SNPs showed significant associations with AIA in our GWAS results (*P*<0.0001; Table 4). Two SNPs, rs139719 and rs7744030, were identified as intergenic SNPs during the first stage of GWAS (build 126 of dbSNP database version), but they have been recently listed as intronic SNPs (build 130 of dbSNP). On the other hand, as for other nearby potential genes in the region where *CEP68* is located, the *RAB1A* gene, despite the lack of reports showing direct relations with aspirin and/or respiratory disease, was also found to be associated with AIA by composing a strong LD with *CEP68* (*P*<0.01; Figure 1).

**Table 4:** Intergenic SNPs with lowest *P* value for risk of aspirin intolerance in the first stage of GWAS.

SNP ID	Variation	Build 126		Build 130	MAF		*P* value*
		Position	Nearby Gene		AIA (n = 80)	ATA (n = 100)	
rs139719	C>T	Intergenic	*bA9F11.1*	SGSM1	0.268	0.475	1.6×10^−6^
rs7744030	C>T	Intergenic	*ENPP4*	CLIC5	0.081	0.245	1.1×10^−5^
rs7963956	T>G	Intergenic	*FAM19A2*		0.311	0.151	4.0×10^−5^
rs4501026	A>C	Intergenic	*C21orf94*		0.292	0.485	6.0×10^−5^
rs346416	C>A	Intergenic	*IPO11*		0.268	0.440	7.3×10^−5^

*P values of co-dominant model. Build 126 and 130 represent the versions of dbSNP.

MAF, minor allele frequency; AIA, aspirin-intolerant asthma; ATA, aspirin-tolerant asthma.

**Figure 2:**
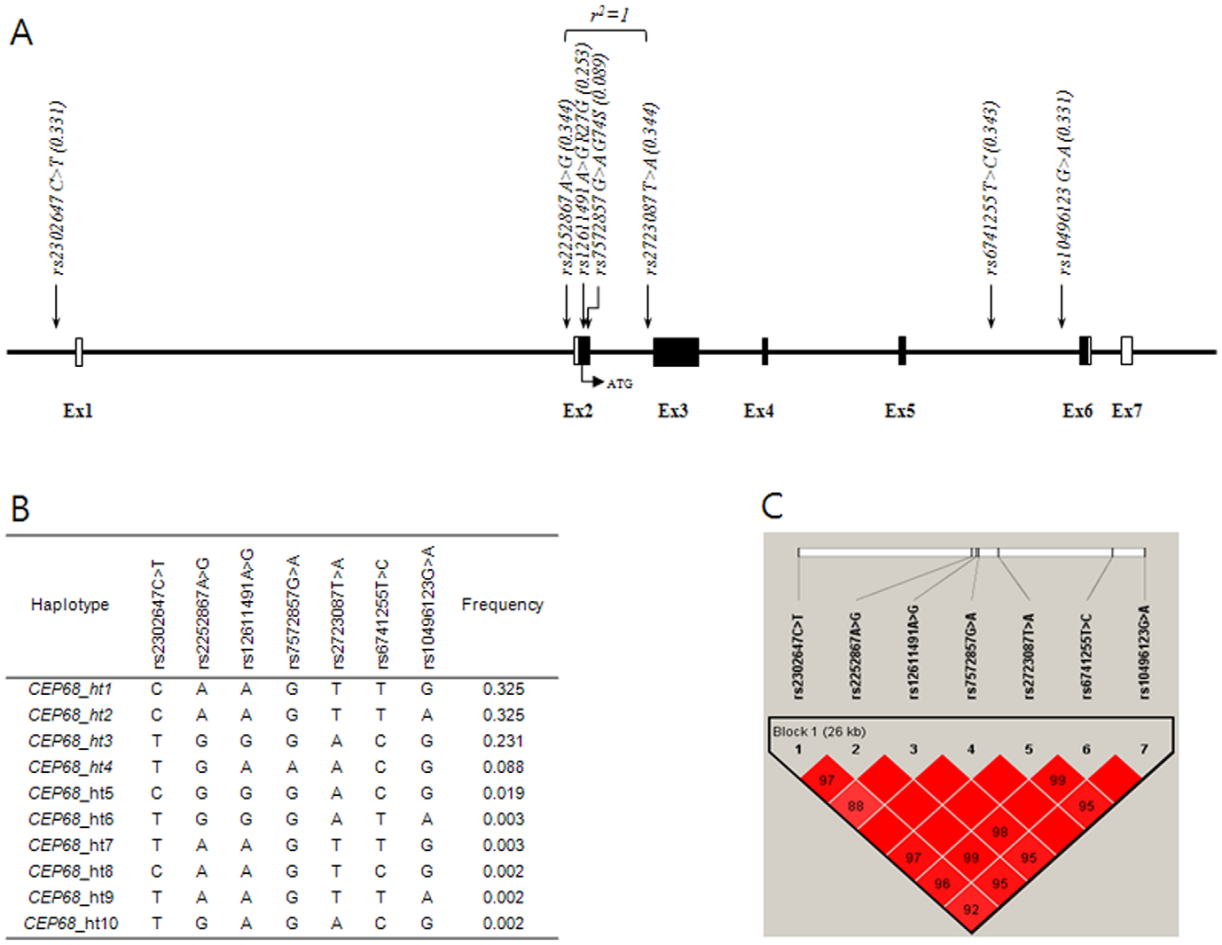
Physical Map, Haplotypes, and LD Plot of *CEP68*. (A) Physical map of *CEP68* and its targeted SNPs. (B) Haplotypes of *CEP68*. Associations of haplotypes with frequency >0.05 are shown in Table 3. (C) LD coefficient (D') among *CEP68* SNPs in a Korean population.

Pair-wise comparisons among SNPs of *CEP68* gene showed tight LDs (Figure 2, Table S4). After haplotypes were inferred using PHASE software, results from logistic analyses for associations between haplotypes and aspirin intolerance showed that haplotype *CEP68_ht4* (T-G-A-A-A-C-G) of AIA was more frequent than that of ATA as a control group, showing that the *CEP68_ht4* with the nonsynonymous rs7572857 “A” allele at the fourth position was significantly associated with AIA in both the co-dominant (OR  = 2.63; 95% CI  = 1.64–4.21; *P* = 6.0×10^−5^, Table 3) and dominant models (OR  = 2.49; 95% CI  = 1.49–4.15; *P* = 0.0005; Table S1) when compared to that of ATA as a control group.

### Effect of rs7572857 on the Decline of FEV_1_ by Aspirin Provocation

**Figure 3:**
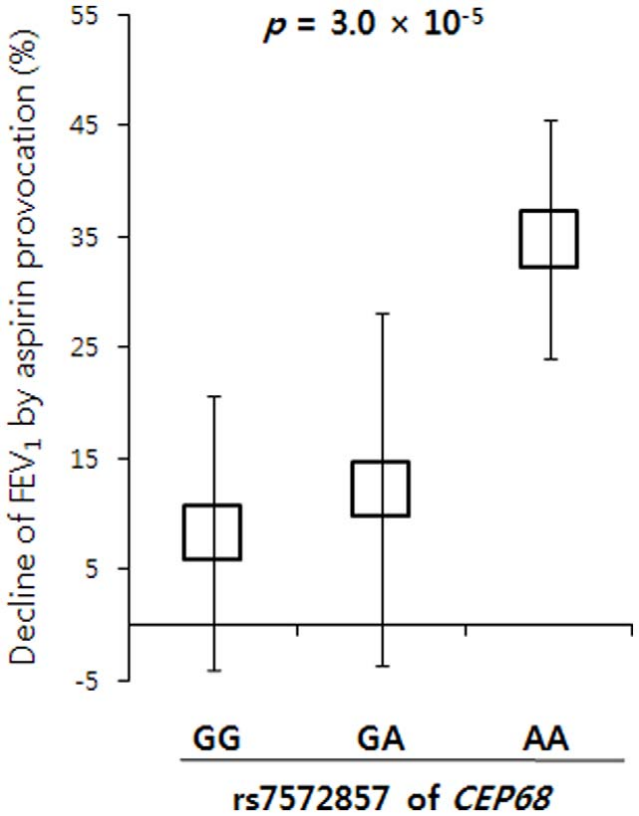
Effect of rs7572857G>A in *CEP68* on the Decline of FEV_1_ by Aspirin Provocation. Distribution presents the mean ± SE values of % decline of FEV_1_ by aspirin provocation in each genotype of the nonsynonymous SNP (rs7572857G>A) in the *CEP68* gene. The homozygote of the rare allele (AA) showed a significant increase in decline of FEV_1_.

In regression analyses, the genotypes of several polymorphisms in the *CEP68* gene were also significantly associated with the decline of FEV_1_ by aspirin provocation. Four SNPs, rs2302647, rs2252867, rs2723087, and rs6741255, showed a similar distribution pattern with about two-fold increase in the mean decline of FEV_1_ for the homozygous rare genotype compared to the homozygous common genotype (Table 5). In the case of rs7572857G>A, however, the nonsynonymous nucleotide substitution showed a different distribution with about a four-fold increase in the mean decline of FEV_1_ for the homozygote of the rare allele (Figure 3), indicating that rs7572857 could have a stronger influence on the higher decline of FEV_1_ by aspirin provocation than other variants. Despite our findings on the relationship between SNP genotypes and decline in FEV_1_ among AIA patients and then among ATA controls, results still showed that rs7572857 has an effect on AIA (*P* = 0.003; Table S5), but not on ATA.

**Table 5:** Association between genotypes of the *CEP68* gene and the decline of FEV_1_ by aspirin provocation.

SNP ID	C/C		C/R		R/R		*P* *
	n	Decline of FEV1 (%)	n	Decline of FEV1 (%)	n	Decline of FEV1 (%)	
rs2302647 C>T	268	7.21±11.22	255	10.00±13.81	69	14.26±16.36	5.0×10^−5^
rs2252867 A>G	258	7.20±11.39	261	9.95±13.69	73	13.85±16.03	9.0×10^−5^
rs12611491 A>G	324	8.20±12.35	233	10.79±14.63	33	8.92±10.11	0.09
rs7572857 G>A	492	8.39±12.34	95	12.26±15.85	5	34.80±10.76	3.0×10^−5^
rs2723087 T>A	258	7.20±11.39	261	9.95±13.69	73	13.85±16.03	9.0×10^−5^
rs6741255 T>C	256	7.20±11.42	266	9.84±13.60	70	14.36±16.16	5.0×10^−5^
rs10496123 G>A	264	10.34±14.28	265	8.55±12.32	63	7.46±12.01	0.06

*P values of regression analyses represent the co-dominant model adjusted for age at initial diagnosis, sex, smoking status and atopy.

C/C, C/R and R/R indicate the homozygote of the common allele, and the heterozygote and homozygote of the rare allele, respectively.

Decline of FEV_1_ represents mean ± SE value.

## Discussion

To our knowledge, this is the first genome-wide association study for AIA. Our results identified *CEP68* as a positive risk factor for the development of aspirin intolerance in asthmatics. Two stages of genome-wide analysis and a follow-up study were used to reduce the number of false-positive SNPs and retain statistical power. In the present study, logistic and regression analyses yielded significant associations between variations of the *CEP68* gene and AIA, with the lowest *P* value at the nonsynonymous SNP rs7572857. In addition, with a tight LD among the SNPs of the *CEP68* gene, the haplotype *CEP68_ht4* (T-G-A-**A**-A-C-G) in AIA subjects was more frequent when compared to that of ATA controls and was significantly associated with AIA. In particular, the fourth positioned “A” that was derived from rs7572857G>A was found only in haplotype *CEP68_ht4*. On the other hand, although we also do not rule out a possible protective effect of rs10496123G>A due to its decreased risk for AIA (OR  = 0.81; Table 3), it is considered that haplotype *CEP68_ht4*, which includes most minor alleles of the significantly associated SNPs and the major G allele of rs10496123G>A, may mainly affect the association with AIA.

Although the inclusion of intermediate AIA in AIA case group also showed significant associations with the SNPs and haplotypes of *CEP68* gene compared to ATA controls, a more correct association for genetic polymorphisms and aspirin intolerance is suggested to the comparison between AIA subjects without intermediate AIA group and ATA controls (Table 3). On the other hand, when observing the effects of polymorphisms of *CEP68* on the decline of FEV_1_ by aspirin provocation, the homozygous AA of rs7572857G>A variant showed a different distribution and a more significant increase of FEV_1_ decline in AIA patients than other variants. In addition, five AIA patients were found to have the homozygote of this rare allele (AA), with 34.80% FEV_1_ decline; whereas no ATA controls were observed to have the AA genotype (Table S5). This observation suggests that the change from glycine to serine could have an effect on the higher decline of FEV_1_ by aspirin provocation.

More recently, there have been debates concerning whether GWAS can successfully detect the variants that are associated with diseases. However, GWAS not only has made it possible to predict risk factors that are associated with diseases, but also has discovered additional variants that are associated with many diseases [34], [35]. Although dense genome-wide genotyping chips containing over 600 K and as many as 1 M SNPs have been recently developed, this study used the earlier chip with about 100 K SNPs due to the starting date of our research. Significantly, ours is the first GWAS to identify genetic factors that might influence the occurrence of aspirin intolerance in asthmatics. On the other hand, while GWAS have found many common variants that have modest effects on human common diseases, albeit some may have a key role, whereas the systematic identification of rare variants that are too rare to be detected in the GWAS and confer a substantial risk of the diseases is also required [36]. In terms of our study's limitations, statistical power indicated that our GWAS contained an insufficient sample size. Statistical powers of 72% in the first GWAS stage and 52% after selecting 150 SNPs in the first stage were estimated by QpowR, an interactive power calculator for two stage association studies [37]. In addition, after Bonferroni correction for multiple comparisons, only two SNPs, rs1053744 in *SBF1* and rs828616 in *DCBLD2*, were significantly associated with AIA. However, this study applied a modest threshold (*P*<0.0001) to select top 11 candidate genes because we focused on the SNPs within a gene region. Moreover, the follow-up study for 11 promising candidate genes increased the power to calculate the association between the *CEP68* gene and aspirin intolerance in asthmatics.

**Figure 4:**
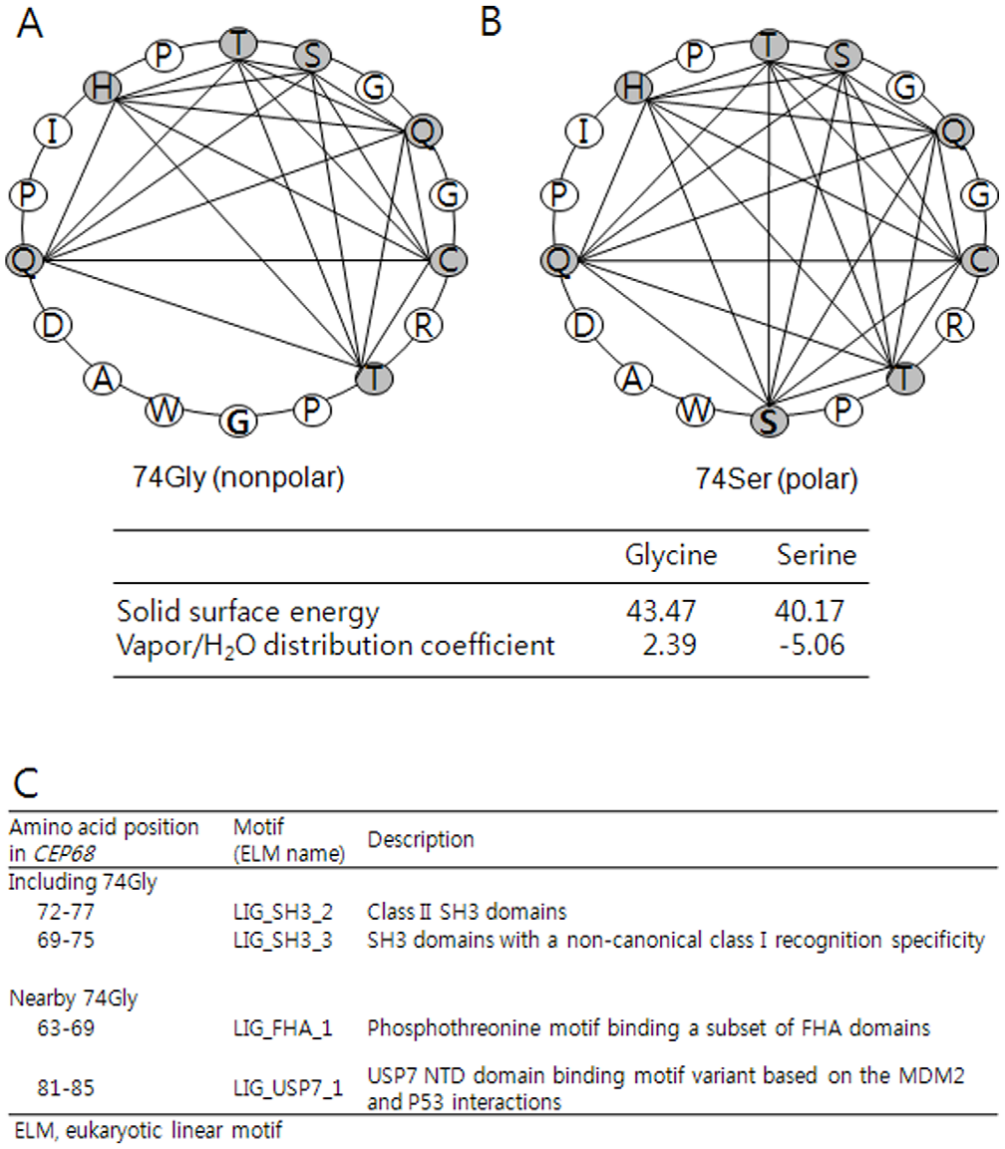
Helical Wheel Projection and Properties between 74Gly and 74Ser, and Motif Search. The helical wheel projection of Gly74Ser is presented by the Expert Protein Analysis System (ExPASy). The change from 74Gly (A) to 74Ser (B) showed an increase in polarity due to its interactions with nearby polar amino acids. The solid surface energy and the degree of vapor/H_2_O distribution of glycine and serine were compared. (C) Motifs including 74Gly and nearby 74Gly for the functional site were found using a prediction program (http://elm.eu.org/index.html).

Since *CEP68* gene (also referred to as *KIAA0582*) in the chromosome 2p14 has been recently discovered, its functions have not been fully understood, except for its role in centrosome cohesion and epidermal growth factor (EGF) signaling [38], [39]. In addition, given that smoke is a risk factor for asthma, *CEP68* has been reported to be differentially expressed following exposure to environmental smoke [40]. However, the functions of the CEP68 protein are not yet fully understood. Thus, our findings provide a new insight into the relationship between *CEP68* and aspirin intolerance in asthma. In particular, it is suggested that the Gly74Ser substitution in exon2 of the *CEP68* gene could affect the polarity of the protein structure (Figure 4A), or affect the function of the CEP68 protein itself [41], [42]. Although *in silico* annotation showed that the nonsynonymous rs7572857G>A (Gly74Ser) appeared to be tolerable to diseases, this site was not highly conserved among mammals (Figure S1). Furthermore, in an additional functional site prediction, it was found that amino acids including 74Gly and its nearby positions could be targets for several kinases (Figure 4B). Since SH domains are especially unique sequences for several regulators, the change from glycine to serine might play an important role in correlations with other modulators. Therefore, phenotypic studies investigating the effects of minor A allele of rs7572857 on protein activity of CEP68 in relation to aspirin sensitivity are needed in the future.

Linkage disequilibrium, as a nonrandom association, has been considered to be profoundly associated with recombination hot spots, quantitative characters, and complex diseases [43]. In addition, experimental evidence has shown an association between haplotype blocks and hot spots of recombination [44]. When compared with other populations from HapMap, the asthmatic subjects in the Korean population showed a stronger LD from the selected *CEP68* SNPs than other populations, with a pattern that was closest to that of the Chinese (Figure S2). Given the significant association of haplotype *CEP68_ht4* with AIA, the LD of *CEP68* might facilitate mapping out genetic factors that function in aspirin metabolism and/or aspirin hypersensitivity in asthma. Therefore, further replications in AIA patients from other populations are required to establish whether or not these structural variations affect AIA.

Other previous reports have suggested associations between genetic polymorphisms and AIA. In one study, 370 SNPs of 63 candidate genes that are involved in the arachidonic acid metabolic cascade were studied for associations with AIA. The results demonstrated that SNPs in the promoter region of the prostaglandin E2 receptor subtype 2 gene were significantly associated with AIA [15]. Several SNPs in the promoter of *LTC4S* [19], [45] and *ALOX5* [20] that encode key enzymes for CysLT synthesis also showed significant associations with AIA [13], [46]. In addition, three promoter polymorphisms in the cysteinyl leukotriene receptor 1 gene were associated with AIA in males [46]. Moreover, polymorphisms of genes that are involved in the pathways of immune response and inflammation are also associated with aspirin hypersensitivity in asthma [21], [25]. This study also confirmed that several other genes, which were associated previously with AIA, showed significant association signals with AIA pathogenesis, especially in the *TNF*, *TGF*, *HLA-DPB1*, *ALOX5* and *IL-10* genes (Table S6). Despite the many studies that have shown positive associations, a comprehensive association between genetic risks and aspirin hypersensitivity has not yet been discovered. Furthermore, constant revisions to find new risk alleles are needed [35], [47]. Hence, our findings on the first genome-wide and follow-up studies of AIA could be a breakthrough in the discovery of susceptible genes using a different approach.

In conclusion, this study confers a positive association between *CEP68* and aspirin-intolerant asthma, suggesting that these findings would be useful for further genetic investigations of NSAIDs metabolism as well as other inflammatory diseases. However, this study does have several limitations. First, this study included only the top 11 genes for the follow-up study due to research budget constraints. Second, our second stage replication analysis was insufficient due to the inclusion of samples from the first stage. In addition, AIA-I patients who were also referred to as positive responders to aspirin hypersensitivity [48], [49] were included in the second round. Although the significances were reduced in the further association analysis without the GWAS subjects due to the high proportion of AIA-I in the second round, the statistically significant association of the SNPs including nonsynonymous rs7572857G>A with AIA still remained (Table S2). Therefore, further replication studies in a larger number of subjects are also needed. Finally, functional studies of the nonsynonymous SNP rs7572857 in the future could provide important insights into the genetic etiology of aspirin intolerance in asthmatics.

## Materials and Methods

### Study Subjects

The subjects were recruited from Soonchunhyang University, Chung Ang University, Chung Nam University, Chungbuk University, and Seoul National University in Korea in a span of about 6 years (between 2003 and 2008). All subjects were Korean. All patients were diagnosed by a physician and met the definition of asthma set forth in the Global Initiative for Asthma guidelines [50]. All patients had a history of dyspnea and wheezing during the previous 12 months, plus one of the following: (1) >15% increase in FEV_1_ or >12% increase plus 200 mL following inhalation of a short-acting bronchodilator, (2) <10 mg/mL PC20 methacholine, and (3) >20% increase in FEV_1_ following 2 weeks of treatment with inhaled steroids and long-acting bronchodilators. Twenty-four common inhalant allergens were used for a skin prick test [26]. Atopy was defined as having a wheal reaction over 3 mm in diameter. Total IgE was measured by the CAP system (Pharmacia Diagnostics, Uppsala, Sweden). The asthmatic patients had experienced no exacerbation of asthma and respiratory tract infection in the 6 weeks preceding the oral aspirin challenge (OAC). The OAC was performed with increasing doses of aspirin using methods slightly modified from those described previously [26], [49]. Changes in FEV_1_ were followed for 5 hours after the last aspirin challenge dose. Aspirin-induced bronchospasms, as reflected by rate (%) of FEV_1_ decline, were calculated as the pre-challenge FEV_1_ minus the post-challenge FEV_1_ divided by the pre-challenge FEV_1_. OAC reactions were categorized into three groups as follows: Subjects with 20% or greater decreases in FEV_1_ or 15% to 19% decreases in FEV_1_ with naso-ocular or cutaneous reactions were designated as the AIA group, those with 15% to 19% decreases in FEV_1_ or naso-ocular or cutaneous reactions only were the AIA-I group, and those with less than 15% decreases in FEV_1_ without naso-ocular or cutaneous reactions were the ATA group. The distribution of FEV_1_ decline rate of subjects is shown in Figure S3. All subjects provided informed consent, and the protocols were approved by the Institutional Review Board of each hospital.

### The First Stage of Genome-Wide SNP Genotyping

About 750 ng of genomic DNA from 80 AIA cases and 100 ATA controls was used to genotype each sample on the Illumina's Human-1 Genotyping BeadChip (Illumina, San Diego, USA). Genotyping was performed according to Illumina's assay manual. Briefly, each sample was processed by whole-genome amplification, fragmentation, precipitation, and resuspension in an appropriate hybridization buffer. Denatured samples were hybridized on the prepared Human-1 Genotyping BeadChip for 16 h at 48°C. Then, the processed beadchip for the single-base extension reaction was stained and imaged on an Illumina Bead Array Reader. To convert fluorescent intensities into SNP genotypes, normalized bead intensity data obtained for each sample were loaded into the Beadstudio 3.0® software (Illumina). SNP clusters for genotype calling of all SNPs were examined using Beadstudio 3.0® software. The overall call rate for all SNPs was 98.0%.

### The Second Stage of Follow-up Study

Eleven genes that include the GWAS-driven top SNPs, which are positioned not at intergenic region but at the 5′-untranslated region (UTR), exons, introns, and the 3′-UTR region, were selected for the follow-up study. For the next study, a total of 150 common SNPs within 11 candidate genes, including nearby upstream region (1.5 kb) of each gene, (8 SNPs for the *SBF1* gene; 12 for *DCBLD2*; 15 for *WDR21A*; 34 for *FILIP1*; 2 for *PDZK3*; 15 for *LRRC43*; 18 for *CIITA*; 5 for *DAF*; 7 for *ENPP5*; 7 for *CEP68*; 27 for *C6*, respectively) were genotyped using a total of 163 AIA cases including AIA-I subjects and 429 ATA controls. The SNPs were scanned using BeadExpress® (Illumina, San Diego, USA).

### Statistics

For genome-wide analysis, associations of genotype distributions between AIA cases and ATA controls were calculated by logistic analyses. In addition to the genetic homogeneity of the study subjects as described above, a parameter (λ) of genomic control was calculated in this study by dividing median χ^2^ statistics by 0.456, with an estimate of 1.056 [51]. Power calculations of the first GWAS stage and after selecting candidate SNPs in the first stage were estimated using QpowR program (https://www.msu.edu/~steibelj/JP_files/QpowR.pdf). In the association analysis of the polymorphisms in *CEP68*, we examined Lewontin's D' (|*D*'|) and the LD coefficient *r^2^* between all pairs of biallelic loci [52]. Haploview v4.1 software downloaded from the Broad Institute (http://www.broadinstitute.org/mpg/haploview) was used to determine LD of *CEP68* [53]. Haplotypes were first estimated using the PHASE software [54], and then computed by logistic analyses using the Statistical Analysis System (SAS) program. Subjects harboring missing genotypes were omitted in the analysis of individual SNPs and haplotypes. Comparisons of genotype distributions between AIA and ATA were carried out with logistic analyses adjusted for initial diagnosed age, sex, smoking status, atopy, and body mass index as multivariates using SAS. The common (C) alleles were used as the referent genotype to the heterozygote and homozygote of the rare (R) allele. The effective numbers of independent marker loci in each gene were calculated to correct for multiple testing using the software SNPSpD (http://genepi.qimr.edu.au/general/daleN/SNPSpD/), which is based on the spectral decomposition (SpD) of matrices of pair-wise LD between SNPs. Significant associations were represented by *P* value <0.05.

## Supporting Information

Table S1 Comparison of co-dominant, dominant and recessive models for SNPs in the CEP68 gene between AIA and ATA.
(0.07 MB DOC)

Table S2 Association analysis without GWAS samples in the second replication round analysis.
(0.05 MB DOC)

Table S3 Logistic analyses of 150 SNPs in Top 11 genes between AIA and ATA.
(0.34 MB DOC)

Table S4 LD coefficients (D' and r2) among CEP68 polymorphisms.
(0.03 MB DOC)

Table S5 Relation of the SNP genotypes in CEP68 to the decline on FEV1 in AIA and in ATA.
(0.05 MB DOC)

Table S6 List of other genes that were associated previously with AIA.
(0.05 MB DOC)

Figure S1 In silico annotation of nonsynonymous rs7572857G>A (Gly74Ser). Impact on protein function and conservation across species are predicted by the SNPs3D program (http://www.snps3d.org/). (A) The higher scores of entropy and Position Specific Scoring Matrix (PSSM) indicate more tolerable to diseases. (B) The 74th amino acid of CEP68 is not highly conserved among mammals.
(0.17 MB DOC)

Figure S2 Comparison of LD from other populations for the selected CEP68 SNPs. The LDs are constructed by seven CEP68 SNPs that are equivalent with this study from HapMap (http://hapmap.ncbi.nlm.nih.gov/index.html.en). CEU, Caucasian; CHB, Chinese, JPT, Japanese; YRI, African.
(0.12 MB DOC)

Figure S3 Distribution of FEV1 decline rate. Distributions are calculated from the decline rate of FEV1 by aspirin provocation and its number of subjects with 5% intervals. The distribution of AIA patients with FEV1 decline rate less than 15% is due to the responders to naso-ocular or cutaneous reactions.
(0.03 MB DOC)
