# Supplementary material for: Genome-Wide and Follow-Up Studies Identify CEP68 Gene Variants Associated with Risk of Aspirin-Intolerant Asthma
Source: PLoS One. 2010 Nov 3;5(11):e13818. doi: 10.1371/journal.pone.0013818 (PMC2972220; doi:10.1371/journal.pone.0013818)
Supplement: Table S1 — Comparison of co-dominant, dominant and recessive models for SNPs in the CEP68 gene between AIA and ATA. (0.07 MB DOC) [file pone.0013818.s001.doc]

**Table S1. Comparison of co-dominant, dominant and recessive models for SNPs in the *CEP68* gene between AIA and ATA.**

|  | AIA (n = 102) vs. ATA (n = 429) | | | | | | | | |  | | [AIA + AIA-I] (n = 163) vs. ATA (n = 429) | | | | | | | | | |
| --- | --- | --- | --- | --- | --- | --- | --- | --- | --- | --- | --- | --- | --- | --- | --- | --- | --- | --- | --- | --- | --- |
|  | Frequency^a^ | |  | Co-dominant | | Dominant | | Recessive | | |  | | Frequency^a^ | |  | Co-dominant model | | Dominant | | Recessive | |
|  | AIA | ATA |  | OR (95% CI) | *p** | OR (95% CI) | *p** | OR (95% CI) | *p** | |  | | **[AIA + AIA-I]** | ATA |  | OR (95% CI) | *p** | OR (95% CI) | *p** | OR (95% CI) | *p** |
| **SNP** |  |  |  |  |  |  |  |  |  | |  | |  |  |  |  |  |  |  |  |  |
| rs2302647 C>T | 0.456 | 0.309 |  | 1.82 (1.32-2.50) | **2.0 × 10^-4^** | 1.94 (1.22-3.09) | **0.005** | 2.76 (1.54-4.92) | **0.0006** | |  | | 0.399 | 0.309 |  | 1.46 (1.12-1.91) | **0.006** | 1.45 (0.99-2.11) | 0.06 | 2.16 (1.27-3.65) | **0.004** |
| rs2252867 A>G | 0.461 | 0.319 |  | 1.78 (1.30-2.45) | **4.0 × 10^-4^** | 1.95 (1.22-3.12) | **0.005** | 2.56 (1.44-4.55) | **0.001** | |  | | 0.414 | 0.319 |  | 1.50 (1.15-1.96) | **0.003** | 1.53 (1.05-2.24) | **0.03** | 2.09 (1.25-3.51) | **0.005** |
| rs12611491 A>G | 0.284 | 0.241 |  | 1.25 (0.88-1.79) | 0.22 | 1.38 (0.89-2.15) | 0.15 | 1.07 (0.42-2.76) | 0.88 | |  | | 0.285 | 0.241 |  | 1.28 (0.95-1.74) | 0.10 | 1.36 (0.94-1.98) | 0.10 | 1.32 (0.62-2.83) | 0.47 |
| rs7572857 G>A | 0.176 | 0.077 |  | 2.63 (1.64-4.21) | **6.0 × 10^-5^** | 2.49 (1.49-4.15) | **0.0005** | - | - | |  | | 0.126 | 0.077 |  | 1.71 (1.11-2.62) | **0.02** | 1.57 (0.99-2.51) | 0.06 | - | - |
| rs2723087 T>A | 0.461 | 0.319 |  | 1.78 (1.30-2.45) | **4.0 × 10^-4^** | 1.95 (1.22-3.12) | **0.005** | 2.56 (1.44-4.55) | **0.001** | |  | | 0.414 | 0.319 |  | 1.50 (1.15-1.96) | **0.003** | 1.53 (1.05-2.24) | **0.03** | 2.09 (1.25-3.51) | **0.005** |
| rs6741255 T>C | 0.466 | 0.317 |  | 1.87 (1.35-2.57) | **1.0 × 10^-4^** | 2.04 (1.27-3.28) | **0.003** | 2.74 (1.54-4.90) | **0.0007** | |  | | 0.417 | 0.317 |  | 1.55 (1.18-2.03) | **0.002** | 1.57 (1.08-2.30) | **0.02** | 2.26 (1.34-3.82) | **0.002** |
| rs10496123 G>A | 0.289 | 0.339 |  | 0.81 (0.58-1.14) | 0.23 | 0.79 (0.51-1.23) | 0.29 | 0.70 (0.33-1.50) | 0.36 | |  | | 0.304 | 0.339 |  | 0.85 (0.64-1.13) | 0.27 | 0.89 (0.61-1.29) | 0.54 | 0.64 (0.33-1.23) | 0.18 |
|  |  |  |  |  |  |  |  |  |  | |  | |  |  |  |  |  |  |  |  |  |
| **Haplotype** |  |  |  |  |  |  |  |  |  | |  | |  |  |  |  |  |  |  |  |  |
| CEP68_*ht1* | 0.240 | 0.340 |  | 0.60 (0.42-0.86) | **0.006** | 0.55 (0.35-0.85) | **0.008** | 0.47 (0.19-1.15) | 0.10 | |  | | 0.276 | 0.340 |  | 0.73 (0.54-0.97) | **0.03** | 0.67 (0.46-0.97) | **0.03** | 0.67 (0.35-1.28) | 0.23 |
| CEP68_*ht2* | 0.289 | 0.331 |  | 0.84 (0.60-1.17) | 0.30 | 0.81 (0.52-1.26) | 0.35 | 0.75 (0.35-1.60) | 0.45 | |  | | 0.304 | 0.331 |  | 0.88 (0.66-1.17) | 0.38 | 0.91 (0.63-1.33) | 0.64 | 0.68 (0.35-1.32) | 0.25 |
| CEP68_*ht3* | 0.275 | 0.218 |  | 1.34 (0.93-1.93) | 0.12 | 1.44 (0.93-2.25) | 0.11 | 1.34 (0.51-3.54) | 0.56 | |  | | 0.267 | 0.218 |  | 1.32 (0.97-1.79) | 0.08 | 1.37 (0.94-1.99) | 0.10 | 1.50 (0.66-3.40) | 0.34 |
| CEP68_*ht4* | 0.176 | 0.077 |  | 2.63 (1.64-4.21) | **6.0 × 10^-5^** | 2.49 (1.49-4.15) | **0.0005** | - | - | |  | | 0.126 | 0.077 |  | 1.71 (1.11-2.62) | **0.02** | 1.57 (0.99-2.51) | 0.06 | - | - |

^a^Frequency indicates the minor allele frequency for SNP; the frequency for haplotype.

**P* values were adjusted for age at initial diagnosis, sex, smoking status, atopy and body mass index.

AIA, aspirin-intolerant asthma; AIA-I, intermediate aspirin-intolerant asthma; ATA, aspirin-tolerant asthma; OR, odds ratio; CI, confidence interval.
