# Supplementary material for: Genome-Wide and Follow-Up Studies Identify CEP68 Gene Variants Associated with Risk of Aspirin-Intolerant Asthma
Source: PLoS One. 2010 Nov 3;5(11):e13818. doi: 10.1371/journal.pone.0013818 (PMC2972220; doi:10.1371/journal.pone.0013818)
Supplement: Table S2 — Association analysis without GWAS samples in the second replication round analysis. (0.05 MB DOC) [file pone.0013818.s002.doc]

**Table S2.** Association analysis without GWAS samples in the second replication round analysis

|  | Frequency^a^ without GWAS samples | |  | Co-dominant | | Dominant | | Recessive | |
| --- | --- | --- | --- | --- | --- | --- | --- | --- | --- |
|  | AIA | ATA |  | OR (95% CI) | *p** | OR (95% CI) | *p** | OR (95% CI) | *p** |
| **SNP** |  |  |  |  |  |  |  |  |  |
| rs2302647 C>T | 0.403 | 0.328 |  | 1.35 (0.94-1.95) | 0.109 | 1.17 (0.70-1.95) | 0.562 | 2.34 (1.18-4.64) | **0.015** |
| rs2252867 A>G | 0.416 | 0.340 |  | 1.37 (0.95-1.98) | 0.090 | 1.18 (0.70-1.99) | 0.528 | 2.35 (1.21-4.60) | **0.012** |
| rs12611491 A>G | 0.273 | 0.261 |  | 1.07 (0.71-1.62) | 0.743 | 1.02 (0.61-1.69) | 0.943 | 1.41 (0.53-3.74) | 0.495 |
| rs7572857 G>A | 0.143 | 0.081 |  | 1.91 (1.08-3.35) | **0.025** | 1.76 (0.95-3.25) | 0.073 | − | − |
| rs2723087 T>A | 0.416 | 0.340 |  | 1.37 (0.95-1.98) | 0.090 | 1.18 (0.70-1.99) | 0.528 | 2.35 (1.21-4.60) | **0.012** |
| rs6741255 T>C | 0.416 | 0.334 |  | 1.41 (0.97-2.03) | 0.071 | 1.19 (0.71-2.00) | 0.512 | 2.54 (1.29-4.98) | **0.007** |
| rs10496123 G>A | 0.312 | 0.334 |  | 0.93 (0.63-1.38) | 0.728 | 1.02 (0.61-1.71) | 0.934 | 0.66 (0.26-1.66) | 0.380 |
|  |  |  |  |  |  |  |  |  |  |
| **Haplotype** |  |  |  |  |  |  |  |  |  |
| CEP68_*ht1* | 0.266 | 0.327 |  | 0.73 (0.49-1.09) | 0.122 | 0.64 (0.38-1.07) | 0.091 | 0.76 (0.32-1.80) | 0.525 |
| CEP68_*ht2* | 0.312 | 0.324 |  | 0.97 (0.65-1.43) | 0.857 | 1.05 (0.63-1.76) | 0.850 | 0.72 (0.28-1.81) | 0.478 |
| CEP68_*ht3* | 0.253 | 0.233 |  | 1.09 (0.71-1.66) | 0.703 | 1.04 (0.62-1.73) | 0.889 | 1.49 (0.50-4.39) | 0.472 |
| CEP68_*ht4* | 0.143 | 0.081 |  | 1.91 (1.08-3.35) | **0.025** | 1.76 (0.95-3.25) | 0.073 | − | − |

^a^Frequency indicates the minor allele frequency for SNP; the frequency for haplotype.

**P* values were adjusted for age at initial diagnosis, sex, smoking status, atopy and body mass index.

AIA, aspirin-intolerant asthma; ATA, aspirin-tolerant asthma; OR, odds ratio; CI, confidence interval.
