# Supplementary material for: Genome-Wide and Follow-Up Studies Identify CEP68 Gene Variants Associated with Risk of Aspirin-Intolerant Asthma
Source: PLoS One. 2010 Nov 3;5(11):e13818. doi: 10.1371/journal.pone.0013818 (PMC2972220; doi:10.1371/journal.pone.0013818)
Supplement: Table S3 — Logistic analyses of 150 SNPs in Top 11 genes between AIA and ATA. (0.34 MB DOC) [file pone.0013818.s003.doc]

**Table S3. Logistic analyses of 150 SNPs in Top 11 genes between AIA and ATA.**

| **Gene** | **SNP ID** | **Position** | **Amino acid change** | **MAF** | | **Co-dominant** | |  | **MAF** | | **Co-dominant** | |
| --- | --- | --- | --- | --- | --- | --- | --- | --- | --- | --- | --- | --- |
|  |  |  |  | **AIA**  **(n=102)** | **ATA**  **(n=429)** | **OR (95% CI)** | ***P**** |  | **[AIA + AIA-I]**  **(n=163)** | **ATA**  **(n=429)** | **OR (95% CI)** | ***P**** |
| *SBF1* | rs738683 G>A | Intron1 |  | 0.206 | 0.152 | 1.40(0.94-2.09) | 0.10 |  | 0.166 | 0.152 | 1.07(0.75-1.53) | 0.70 |
|  | rs2076714 T>C | Intron13 |  | 0.534 | 0.491 | 1.14(0.83-1.57) | 0.43 |  | 0.509 | 0.491 | 1.04(0.80-1.36) | 0.77 |
|  | rs5771040 G>A | Intron15 |  | 0.049 | 0.044 | 1.10(0.52-2.32) | 0.81 |  | 0.037 | 0.044 | 0.79(0.40-1.58) | 0.50 |
|  | rs2236030 G>A | Intron23 |  | 0.402 | 0.391 | 1.10(0.79-1.51) | 0.58 |  | 0.408 | 0.391 | 1.09(0.84-1.43) | 0.51 |
|  | rs2073277 C>T | Intron26 |  | 0.059 | 0.044 | 1.33(0.69-2.56) | 0.39 |  | 0.055 | 0.044 | 1.25(0.71-2.23) | 0.44 |
|  | rs4824149 G>A | Intron28 |  | 0.277 | 0.316 | 0.80(0.56-1.15) | 0.23 |  | 0.293 | 0.316 | 0.89(0.67-1.20) | 0.45 |
|  | rs9617010 G>C | Intron35 |  | 0.356 | 0.418 | 0.75(0.54-1.06) | 0.10 |  | 0.386 | 0.418 | 0.88(0.67-1.16) | 0.37 |
|  | rs1053744 C>T | Exon39 | T1827T | 0.505 | 0.469 | 1.17(0.86-1.60) | 0.32 |  | 0.506 | 0.469 | 1.16(0.88-1.51) | 0.29 |
| *DCBLD2* | rs2439224 T>C | Intron1 |  | 0.113 | 0.170 | 0.63(0.39-1.02) | 0.06 |  | 0.129 | 0.170 | 0.74(0.51-1.07) | 0.11 |
|  | rs1371687 C>T | Intron1 |  | 0.265 | 0.334 | 0.73(0.51-1.05) | 0.09 |  | 0.267 | 0.334 | 0.75(0.56-1.00) | 0.05 |
|  | rs9838238 T>C | Exon2 | I144M | 0.025 | 0.030 | 0.82(0.30-2.25) | 0.70 |  | 0.025 | 0.030 | 0.83(0.36-1.91) | 0.66 |
|  | rs17278047 G>C | Intron2 |  | 0.054 | 0.068 | 0.93(0.48-1.78) | 0.82 |  | 0.052 | 0.068 | 0.89(0.51-1.55) | 0.68 |
|  | rs7615856 T>C | Intron2 |  | 0.240 | 0.304 | 0.74(0.51-1.07) | 0.11 |  | 0.242 | 0.304 | 0.75(0.56-1.02) | 0.06 |
|  | rs828621 A>T | Intron4 |  | 0.240 | 0.305 | 0.74(0.51-1.06) | 0.10 |  | 0.242 | 0.305 | 0.75(0.56-1.01) | 0.06 |
|  | rs828618 G>A | Intron4 |  | 0.510 | 0.409 | 1.50(1.08-2.09) | **0.02** |  | 0.482 | 0.409 | 1.31(1.00-1.71) | 0.05 |
|  | rs828616 A>G | Exon6 | I262I | 0.265 | 0.337 | 0.73(0.51-1.04) | 0.08 |  | 0.267 | 0.337 | 0.74(0.55-0.99) | **0.04** |
|  | rs16840208 C>T | Exon16 | D723N | 0.025 | 0.062 | 0.39(0.15-1.00) | 0.05 |  | 0.040 | 0.062 | 0.66(0.35-1.23) | 0.19 |
|  | rs17270986 A>G | 3'UTR |  | 0.113 | 0.167 | 0.64(0.39-1.04) | 0.07 |  | 0.129 | 0.167 | 0.75(0.51-1.09) | 0.13 |
|  | rs1062196 A>G | 3'UTR |  | 0.152 | 0.204 | 0.69(0.45-1.08) | 0.10 |  | 0.163 | 0.204 | 0.76(0.54-1.08) | 0.13 |
|  | rs8833 T>C | 3'UTR |  | 0.225 | 0.302 | 0.70(0.48-1.03) | 0.07 |  | 0.236 | 0.302 | 0.75(0.56-1.02) | 0.07 |
| *WDR21A* | rs10483851 C>T | Promoter |  | 0.025 | 0.031 | 0.76(0.29-2.01) | 0.58 |  | 0.028 | 0.031 | 0.89(0.41-1.93) | 0.77 |
|  | rs7142086 C>T | Promoter |  | 0.186 | 0.149 | 1.24(0.82-1.87) | 0.32 |  | 0.181 | 0.149 | 1.20(0.85-1.71) | 0.31 |
|  | rs17781739 G>T | Promoter |  | 0.059 | 0.082 | 0.71(0.37-1.37) | 0.31 |  | 0.064 | 0.082 | 0.79(0.47-1.34) | 0.38 |
|  | rs3213729 C>G | 5'UTR |  | 0.186 | 0.150 | 1.23(0.81-1.86) | 0.33 |  | 0.181 | 0.150 | 1.19(0.84-1.69) | 0.33 |
|  | rs7160796 G>C | Intron1 |  | 0.211 | 0.236 | 0.89(0.62-1.28) | 0.54 |  | 0.212 | 0.236 | 0.90(0.66-1.22) | 0.48 |
|  | rs11158993 C>T | Intron1 |  | 0.044 | 0.070 | 0.62(0.30-1.27) | 0.19 |  | 0.055 | 0.070 | 0.77(0.45-1.34) | 0.36 |
|  | rs12892806 C>G | Intron1 |  | 0.412 | 0.364 | 1.27(0.92-1.75) | 0.15 |  | 0.374 | 0.364 | 1.06(0.81-1.40) | 0.66 |
|  | rs2302588 C>G | Exon2 | C22W | 0.152 | 0.136 | 1.11(0.71-1.74) | 0.64 |  | 0.141 | 0.136 | 0.99(0.68-1.45) | 0.95 |
|  | rs12885397 G>A | Intron4 |  | 0.230 | 0.214 | 1.16(0.80-1.69) | 0.44 |  | 0.214 | 0.214 | 1.07(0.77-1.47) | 0.70 |
|  | rs3742832 T>C | Intron4 |  | 0.456 | 0.484 | 0.89(0.65-1.21) | 0.46 |  | 0.488 | 0.484 | 1.03(0.79-1.33) | 0.85 |
|  | rs2240981 C>T | Intron6 |  | 0.422 | 0.472 | 0.80(0.58-1.11) | 0.18 |  | 0.457 | 0.472 | 0.95(0.73-1.24) | 0.70 |
|  | rs2286833 G>A | Intron7 |  | 0.064 | 0.059 | 1.16(0.59-2.28) | 0.66 |  | 0.067 | 0.059 | 1.22(0.70-2.12) | 0.48 |
|  | rs2806039 G>A | Intron8 |  | 0.186 | 0.162 | 1.24(0.82-1.87) | 0.31 |  | 0.169 | 0.162 | 1.11(0.78-1.58) | 0.57 |
|  | rs7143683 T>C | Intron13 |  | 0.515 | 0.470 | 1.19(0.87-1.62) | 0.27 |  | 0.475 | 0.470 | 1.01(0.78-1.30) | 0.97 |
|  | rs7144738 A>C | 3'UTR |  | 0.206 | 0.171 | 1.32(0.88-1.96) | 0.18 |  | 0.181 | 0.171 | 1.13(0.80-1.59) | 0.49 |
| *FILIP1* | rs9343292 A>G | Intron1 |  | 0.118 | 0.118 | 0.96(0.59-1.56) | 0.87 |  | 0.126 | 0.118 | 1.04(0.70-1.55) | 0.84 |
|  | rs17496664 C>T | Intron1 |  | 0.025 | 0.040 | 0.56(0.21-1.50) | 0.25 |  | 0.028 | 0.040 | 0.60(0.28-1.31) | 0.20 |
|  | rs1932523 T>C | Intron1 |  | 0.382 | 0.347 | 1.21(0.87-1.69) | 0.26 |  | 0.402 | 0.347 | 1.30(0.98-1.72) | 0.07 |
|  | rs10806039 A>G | Intron1 |  | 0.368 | 0.334 | 1.21(0.86-1.69) | 0.28 |  | 0.393 | 0.334 | 1.33(1.00-1.76) | 0.05 |
|  | rs12211255 C>A | Intron1 |  | 0.113 | 0.117 | 0.92(0.55-1.52) | 0.73 |  | 0.126 | 0.117 | 1.06(0.71-1.58) | 0.79 |
|  | rs9360910 T>G | Intron1 |  | 0.230 | 0.178 | 1.55(1.04-2.29) | **0.03** |  | 0.239 | 0.178 | 1.59(1.14-2.21) | **0.006** |
|  | rs2951916 T>C | Intron1 |  | 0.304 | 0.294 | 0.99(0.71-1.39) | 0.95 |  | 0.322 | 0.294 | 1.10(0.82-1.46) | 0.53 |
|  | rs11754006 T>G | Intron1 |  | 0.137 | 0.160 | 0.79(0.50-1.24) | 0.31 |  | 0.153 | 0.160 | 0.90(0.62-1.30) | 0.57 |
|  | rs4594918 G>A | Intron1 |  | 0.368 | 0.337 | 1.19(0.85-1.66) | 0.32 |  | 0.393 | 0.337 | 1.31(0.99-1.74) | 0.06 |
|  | rs2951918 G>T | Intron1 |  | 0.382 | 0.350 | 1.19(0.85-1.67) | 0.30 |  | 0.402 | 0.350 | 1.28(0.97-1.69) | 0.08 |
|  | rs2808182 T>G | Intron1 |  | 0.466 | 0.524 | 0.77(0.56-1.07) | 0.12 |  | 0.439 | 0.524 | 0.69(0.52-0.91) | **0.008** |
|  | rs12209223 C>A | Intron1 |  | 0.103 | 0.107 | 0.86(0.51-1.46) | 0.58 |  | 0.120 | 0.107 | 1.05(0.70-1.59) | 0.82 |
|  | rs2808181 T>G | Intron1 |  | 0.230 | 0.203 | 1.13(0.78-1.64) | 0.53 |  | 0.242 | 0.203 | 1.19(0.87-1.61) | 0.28 |
|  | rs2998394 A>C | Intron1 |  | 0.417 | 0.490 | 0.74(0.53-1.02) | 0.07 |  | 0.405 | 0.490 | 0.70(0.53-0.92) | **0.01** |
|  | rs2998393 G>A | Intron1 |  | 0.431 | 0.386 | 1.25(0.90-1.73) | 0.19 |  | 0.436 | 0.386 | 1.25(0.95-1.64) | 0.12 |
|  | rs2951932 C>T | Intron1 |  | 0.417 | 0.488 | 0.74(0.53-1.02) | 0.07 |  | 0.405 | 0.488 | 0.70(0.53-0.92) | **0.01** |
|  | rs2951929 G>A | Intron1 |  | 0.416 | 0.488 | 0.74(0.53-1.02) | 0.07 |  | 0.404 | 0.488 | 0.70(0.53-0.92) | **0.01** |
|  | rs9447478 G>C | Intron1 |  | 0.186 | 0.169 | 1.24(0.81-1.88) | 0.32 |  | 0.184 | 0.169 | 1.20(0.84-1.70) | 0.31 |
|  | rs9447476 C>G | Intron1 |  | 0.201 | 0.183 | 1.23(0.82-1.84) | 0.32 |  | 0.193 | 0.183 | 1.15(0.82-1.61) | 0.43 |
|  | rs7771904 A>G | Intron2 |  | 0.412 | 0.392 | 1.11(0.80-1.53) | 0.55 |  | 0.414 | 0.392 | 1.08(0.83-1.42) | 0.56 |
|  | rs2842457 T>C | Intron2 |  | 0.142 | 0.122 | 1.16(0.73-1.82) | 0.53 |  | 0.153 | 0.122 | 1.32(0.91-1.93) | 0.15 |
|  | rs9293999 G>A | Intron2 |  | 0.201 | 0.203 | 0.95(0.65-1.40) | 0.80 |  | 0.190 | 0.203 | 0.87(0.63-1.21) | 0.42 |
|  | rs4616955 C>A | Intron2 |  | 0.108 | 0.098 | 1.27(0.75-2.14) | 0.38 |  | 0.110 | 0.098 | 1.28(0.83-1.98) | 0.27 |
|  | rs2703710 C>T | Intron2 |  | 0.142 | 0.122 | 1.16(0.73-1.82) | 0.53 |  | 0.150 | 0.122 | 1.29(0.88-1.89) | 0.19 |
|  | rs2951952 A>G | Intron3 |  | 0.441 | 0.485 | 0.84(0.61-1.15) | 0.28 |  | 0.433 | 0.485 | 0.82(0.63-1.07) | 0.14 |
|  | rs2951953 C>T | Intron3 |  | 0.142 | 0.124 | 1.15(0.73-1.81) | 0.55 |  | 0.150 | 0.124 | 1.28(0.88-1.88) | 0.20 |
|  | rs2951934 A>G | Intron4 |  | 0.441 | 0.485 | 0.84(0.61-1.15) | 0.28 |  | 0.433 | 0.485 | 0.82(0.63-1.07) | 0.14 |
|  | rs4324739 T>G | Intron4 |  | 0.441 | 0.484 | 0.84(0.61-1.16) | 0.28 |  | 0.433 | 0.484 | 0.82(0.63-1.07) | 0.14 |

**Table S3.** Continued.

|  | rs4579317 A>T | Intron4 |  | 0.230 | 0.216 | 1.04(0.72-1.51) | 0.84 |  | 0.227 | 0.216 | 1.00(0.73-1.36) | 0.99 |
| --- | --- | --- | --- | --- | --- | --- | --- | --- | --- | --- | --- | --- |
|  | rs9341514 A>G | Intron4 |  | 0.417 | 0.393 | 1.12(0.81-1.55) | 0.50 |  | 0.417 | 0.393 | 1.09(0.83-1.42) | 0.54 |
|  | rs16886475 C>A | Intron4 |  | 0.137 | 0.142 | 1.06(0.67-1.68) | 0.81 |  | 0.147 | 0.142 | 1.13(0.78-1.65) | 0.52 |
|  | rs4131550 T>G | Intron4 |  | 0.417 | 0.393 | 1.12(0.81-1.55) | 0.50 |  | 0.417 | 0.393 | 1.09(0.83-1.42) | 0.54 |
|  | rs7743227 A>C | Intron4 |  | 0.387 | 0.353 | 1.18(0.85-1.65) | 0.32 |  | 0.387 | 0.353 | 1.15(0.88-1.52) | 0.31 |
|  | rs4708179 C>T | Intron4 |  | 0.387 | 0.353 | 1.18(0.85-1.65) | 0.32 |  | 0.387 | 0.353 | 1.15(0.88-1.52) | 0.31 |
| *PDZK3* | rs4867084 G>A | Intron1 |  | 0.201 | 0.296 | 0.59(0.40-0.86) | **0.006** |  | 0.233 | 0.296 | 0.71(0.52-0.95) | **0.02** |
|  | rs10066063 G>A | Exon19 | R2247Q | 0.233 | 0.250 | 0.86(0.59-1.26) | 0.44 |  | 0.238 | 0.250 | 0.91(0.66-1.25) | 0.57 |
| *LRRC43* | rs11058785 G>A | Promoter |  | 0.074 | 0.100 | 0.65(0.36-1.16) | 0.15 |  | 0.092 | 0.100 | 0.81(0.52-1.27) | 0.36 |
|  | rs11058789 T>C | Promoter |  | 0.270 | 0.317 | 0.74(0.52-1.05) | 0.09 |  | 0.291 | 0.317 | 0.84(0.63-1.12) | 0.23 |
|  | rs4758680 C>A | Intron1 |  | 0.196 | 0.218 | 0.83(0.56-1.24) | 0.36 |  | 0.199 | 0.218 | 0.88(0.63-1.23) | 0.45 |
|  | rs7977932 C>G | Intron1 |  | 0.074 | 0.103 | 0.64(0.35-1.14) | 0.13 |  | 0.092 | 0.103 | 0.80(0.51-1.25) | 0.32 |
|  | rs10847385 A>C | Intron1 |  | 0.079 | 0.108 | 0.66(0.38-1.17) | 0.15 |  | 0.102 | 0.108 | 0.86(0.56-1.31) | 0.47 |
|  | rs12828783 C>T | Intron1 |  | 0.265 | 0.328 | 0.68(0.48-0.97) | **0.03** |  | 0.288 | 0.328 | 0.78(0.58-1.05) | 0.10 |
|  | rs2292443 G>A | Intron1 |  | 0.196 | 0.226 | 0.79(0.53-1.17) | 0.23 |  | 0.199 | 0.226 | 0.83(0.60-1.16) | 0.27 |
|  | rs7302430 G>A | Intron2 |  | 0.069 | 0.089 | 0.68(0.37-1.25) | 0.21 |  | 0.089 | 0.089 | 0.88(0.56-1.40) | 0.60 |
|  | rs11060094 C>A | Exon7 | Q127K | 0.020 | 0.035 | 0.48(0.16-1.41) | 0.18 |  | 0.022 | 0.035 | 0.52(0.22-1.23) | 0.14 |
|  | rs4758677 T>C | Intron7 |  | 0.240 | 0.307 | 0.65(0.45-0.95) | **0.03** |  | 0.267 | 0.307 | 0.76(0.56-1.04) | 0.08 |
|  | rs12810587 T>C | Intron7 |  | 0.218 | 0.271 | 0.69(0.47-1.02) | 0.06 |  | 0.241 | 0.271 | 0.80(0.58-1.09) | 0.15 |
|  | rs11060167 C>A | Exon8 | R201R | 0.240 | 0.312 | 0.63(0.43-0.92) | **0.02** |  | 0.270 | 0.312 | 0.76(0.56-1.03) | 0.07 |
|  | rs10847803 A>G | Intron8 |  | 0.030 | 0.046 | 0.58(0.23-1.42) | 0.23 |  | 0.040 | 0.046 | 0.78(0.40-1.51) | 0.46 |
|  | rs11060333 C>T | Intron9 |  | 0.235 | 0.314 | 0.60(0.41-0.88) | **0.009** |  | 0.267 | 0.314 | 0.74(0.54-1.00) | 0.05 |
|  | rs872451 G>C | Intron13 |  | 0.235 | 0.304 | 0.64(0.44-0.93) | **0.02** |  | 0.261 | 0.304 | 0.74(0.55-1.00) | 0.05 |
| *CIITA* | rs12932187 C>G | Intron1 |  | 0.422 | 0.389 | 1.09(0.80-1.47) | 0.60 |  | 0.411 | 0.389 | 1.06(0.82-1.37) | 0.65 |
|  | rs4781011 T>G | Intron1 |  | 0.127 | 0.129 | 0.99(0.63-1.56) | 0.95 |  | 0.147 | 0.129 | 1.15(0.80-1.66) | 0.44 |
|  | rs11074934 T>C | Intron1 |  | 0.275 | 0.310 | 0.85(0.61-1.19) | 0.35 |  | 0.301 | 0.310 | 0.95(0.72-1.25) | 0.70 |
|  | rs8043545 C>G | Intron1 |  | 0.480 | 0.473 | 1.07(0.79-1.45) | 0.65 |  | 0.475 | 0.473 | 1.06(0.82-1.36) | 0.68 |
|  | rs8063850 T>A | Intron3 |  | 0.078 | 0.096 | 0.84(0.47-1.48) | 0.54 |  | 0.110 | 0.096 | 1.20(0.78-1.83) | 0.41 |
|  | rs6498119 T>C | Intron3 |  | 0.343 | 0.289 | 1.23(0.89-1.70) | 0.21 |  | 0.331 | 0.289 | 1.18(0.90-1.55) | 0.23 |
|  | rs7189406 A>G | Intron5 |  | 0.436 | 0.459 | 0.93(0.68-1.27) | 0.64 |  | 0.423 | 0.459 | 0.88(0.68-1.15) | 0.36 |
|  | rs6498124 G>T | Intron6 |  | 0.387 | 0.464 | 0.73(0.54-1.00) | **0.05** |  | 0.396 | 0.464 | 0.76(0.58-0.99) | **0.04** |
|  | rs4781016 C>A | Intron7 |  | 0.191 | 0.198 | 0.93(0.62-1.37) | 0.70 |  | 0.193 | 0.198 | 0.94(0.68-1.30) | 0.70 |
|  | rs4774 G>C | Exon11 | G500A | 0.191 | 0.200 | 0.91(0.61-1.35) | 0.62 |  | 0.193 | 0.200 | 0.92(0.66-1.27) | 0.60 |
|  | rs4781019 C>T | Intron13 |  | 0.324 | 0.350 | 0.86(0.61-1.23) | 0.42 |  | 0.322 | 0.350 | 0.87(0.65-1.16) | 0.34 |
|  | rs6498126 C>G | Intron13 |  | 0.270 | 0.291 | 0.91(0.63-1.31) | 0.60 |  | 0.267 | 0.291 | 0.90(0.66-1.22) | 0.49 |
|  | rs11074938 G>A | Intron13 |  | 0.466 | 0.490 | 0.87(0.63-1.20) | 0.40 |  | 0.479 | 0.490 | 0.92(0.70-1.20) | 0.54 |
|  | rs11074939 G>A | Intron15 |  | 0.358 | 0.343 | 1.06(0.76-1.47) | 0.75 |  | 0.347 | 0.343 | 1.01(0.76-1.34) | 0.95 |
|  | rs7404786 C>G | Intron16 |  | 0.196 | 0.192 | 1.04(0.69-1.56) | 0.85 |  | 0.193 | 0.192 | 1.01(0.72-1.41) | 0.96 |
|  | rs7201430 C>T | Exon17 | C1057C | 0.167 | 0.148 | 1.18(0.77-1.83) | 0.45 |  | 0.166 | 0.148 | 1.16(0.81-1.67) | 0.42 |
|  | rs4781024 G>A | Intron18 |  | 0.358 | 0.323 | 1.15(0.83-1.61) | 0.41 |  | 0.340 | 0.323 | 1.06(0.80-1.41) | 0.67 |
|  | rs1139564 C>T | 3'UTR |  | 0.216 | 0.233 | 0.93(0.64-1.35) | 0.70 |  | 0.227 | 0.233 | 0.98(0.72-1.34) | 0.92 |
| *DAF* | rs2564978 T>C | Promoter |  | 0.471 | 0.459 | 1.04(0.76-1.41) | 0.82 |  | 0.485 | 0.459 | 1.10(0.85-1.42) | 0.47 |
|  | rs4844590 C>T | Intron2 |  | 0.064 | 0.059 | 1.05(0.55-1.98) | 0.89 |  | 0.074 | 0.059 | 1.21(0.72-2.02) | 0.48 |
|  | rs6691942 C>T | Intron5 |  | 0.407 | 0.400 | 1.03(0.75-1.40) | 0.87 |  | 0.411 | 0.400 | 1.05(0.81-1.37) | 0.70 |
|  | rs925131 A>G | Intron5 |  | 0.471 | 0.460 | 1.03(0.76-1.41) | 0.84 |  | 0.485 | 0.460 | 1.09(0.85-1.42) | 0.50 |
|  | rs2782837 C>T | Intron9 |  | 0.218 | 0.181 | 1.23(0.84-1.78) | 0.29 |  | 0.204 | 0.181 | 1.14(0.82-1.56) | 0.44 |
| *ENPP5* | rs3806995 C>A | Exon2 | L6I | 0.397 | 0.381 | 1.02(0.74-1.40) | 0.93 |  | 0.408 | 0.381 | 1.08(0.82-1.41) | 0.59 |
|  | rs6926570 T>C | Exon2 | I171V | 0.397 | 0.381 | 1.02(0.74-1.40) | 0.93 |  | 0.408 | 0.381 | 1.08(0.82-1.41) | 0.59 |
|  | rs9472714 G>A | Intron2 |  | 0.132 | 0.143 | 0.88(0.57-1.39) | 0.59 |  | 0.144 | 0.143 | 1.00(0.70-1.44) | 10.00 |
|  | rs16874326 T>C | Exon3 | Y283C | 0.039 | 0.035 | 1.02(0.45-2.28) | 0.97 |  | 0.040 | 0.035 | 1.02(0.51-2.01) | 0.97 |
|  | rs16874322 T>A | Intron3 |  | 0.196 | 0.224 | 0.86(0.58-1.27) | 0.43 |  | 0.202 | 0.224 | 0.89(0.65-1.23) | 0.49 |
|  | rs1047153 A>G | 3'UTR |  | 0.392 | 0.390 | 1.05(0.76-1.43) | 0.78 |  | 0.377 | 0.390 | 0.98(0.75-1.28) | 0.88 |
|  | rs2295017 A>T | 3'UTR |  | 0.132 | 0.143 | 0.88(0.57-1.39) | 0.59 |  | 0.144 | 0.143 | 1.00(0.70-1.44) | 10.00 |
| *CEP68* | rs2302647 C>T | Promoter |  | 0.456 | 0.309 | 1.82(1.32-2.50) | **0.0002** |  | 0.399 | 0.309 | 1.46(1.12-1.91) | **0.006** |
|  | rs2252867 A>G | Intron1 |  | 0.461 | 0.319 | 1.78(1.30-2.45) | **0.0004** |  | 0.414 | 0.319 | 1.50(1.15-1.96) | **0.003** |
|  | rs12611491 A>G | Exon2 | R27G | 0.284 | 0.241 | 1.25(0.88-1.79) | 0.22 |  | 0.285 | 0.241 | 1.28(0.95-1.74) | 0.10 |
|  | rs7572857 G>A | Exon2 | G74S | 0.176 | 0.077 | 2.63(1.64-4.21) | **0.00006** |  | 0.126 | 0.077 | 1.71(1.11-2.62) | **0.02** |
|  | rs2723087 T>A | Intron2 |  | 0.461 | 0.319 | 1.78(1.30-2.45) | **0.0004** |  | 0.414 | 0.319 | 1.50(1.15-1.96) | **0.003** |
|  | rs6741255 T>C | Intron5 |  | 0.466 | 0.317 | 1.87(1.35-2.57) | **0.0001** |  | 0.417 | 0.317 | 1.55(1.18-2.03) | **0.002** |
|  | rs10496123 G>A | Intron5 |  | 0.289 | 0.339 | 0.81(0.58-1.14) | 0.23 |  | 0.304 | 0.339 | 0.85(0.64-1.13) | 0.27 |
| *C6* | rs2921174 G>T | Promoter |  | 0.198 | 0.161 | 1.34(0.88-2.05) | 0.18 |  | 0.185 | 0.161 | 1.22(0.85-1.74) | 0.29 |
|  | rs10512766 C>G | Intron1 |  | 0.054 | 0.105 | 0.55(0.30-1.02) | 0.06 |  | 0.061 | 0.105 | 0.60(0.37-0.98) | **0.04** |
|  | rs13359770 T>C | Intron1 |  | 0.137 | 0.146 | 0.95(0.62-1.47) | 0.83 |  | 0.129 | 0.146 | 0.89(0.61-1.30) | 0.55 |
|  | rs1822821 G>C | Intron1 |  | 0.402 | 0.344 | 1.28(0.94-1.74) | 0.11 |  | 0.380 | 0.344 | 1.17(0.90-1.51) | 0.25 |
|  | rs1444903 C>T | Intron1 |  | 0.162 | 0.195 | 0.76(0.50-1.14) | 0.18 |  | 0.178 | 0.195 | 0.86(0.62-1.19) | 0.36 |

**Table S3.** Continued.

|  | rs2305060 C>T | 5'UTR |  | 0.137 | 0.146 | 0.95(0.62-1.47) | 0.83 |  | 0.129 | 0.146 | 0.89(0.61-1.30) | 0.55 |
| --- | --- | --- | --- | --- | --- | --- | --- | --- | --- | --- | --- | --- |
|  | rs1801033 C>A | Exon4 | A119E | 0.461 | 0.449 | 1.08(0.79-1.47) | 0.63 |  | 0.445 | 0.449 | 0.99(0.77-1.29) | 0.97 |
|  | rs7443604 G>T | Intron4 |  | 0.461 | 0.449 | 1.08(0.79-1.47) | 0.63 |  | 0.445 | 0.449 | 0.99(0.77-1.29) | 0.97 |
|  | rs6892389 A>G | Intron4 |  | 0.162 | 0.206 | 0.72(0.48-1.08) | 0.11 |  | 0.187 | 0.206 | 0.86(0.62-1.18) | 0.34 |
|  | rs10473238 C>T | Intron4 |  | 0.137 | 0.146 | 0.95(0.61-1.47) | 0.82 |  | 0.129 | 0.146 | 0.89(0.61-1.29) | 0.54 |
|  | rs11951782 T>A | Intron5 |  | 0.201 | 0.161 | 1.37(0.90-2.09) | 0.14 |  | 0.190 | 0.161 | 1.27(0.89-1.81) | 0.19 |
|  | rs4413571 T>A | Intron5 |  | 0.230 | 0.290 | 0.76(0.53-1.08) | 0.12 |  | 0.242 | 0.290 | 0.81(0.60-1.08) | 0.15 |
|  | rs6860770 A>C | Intron6 |  | 0.137 | 0.146 | 0.95(0.61-1.47) | 0.82 |  | 0.129 | 0.146 | 0.89(0.61-1.29) | 0.54 |
|  | rs4245976 C>T | Intron6 |  | 0.206 | 0.162 | 1.41(0.93-2.15) | 0.11 |  | 0.184 | 0.162 | 1.20(0.84-1.72) | 0.33 |
|  | rs11954878 C>T | Intron6 |  | 0.392 | 0.332 | 1.28(0.94-1.74) | 0.11 |  | 0.368 | 0.332 | 1.16(0.90-1.51) | 0.25 |
|  | rs4957375 C>T | Intron9 |  | 0.309 | 0.277 | 1.16(0.83-1.62) | 0.39 |  | 0.282 | 0.277 | 1.01(0.76-1.34) | 0.97 |
|  | rs6451566 C>T | Intron9 |  | 0.319 | 0.275 | 1.24(0.89-1.73) | 0.20 |  | 0.291 | 0.275 | 1.09(0.82-1.46) | 0.54 |
|  | rs3805715 A>G | Intron13 |  | 0.322 | 0.379 | 0.78(0.56-1.07) | 0.12 |  | 0.361 | 0.379 | 0.93(0.72-1.21) | 0.58 |
|  | rs10512764 C>T | Intron13 |  | 0.363 | 0.418 | 0.78(0.57-1.07) | 0.12 |  | 0.411 | 0.418 | 0.97(0.75-1.26) | 0.83 |
|  | rs3805714 T>A | Intron13 |  | 0.039 | 0.040 | 0.94(0.42-2.12) | 0.89 |  | 0.052 | 0.040 | 1.39(0.75-2.60) | 0.30 |
|  | rs2301247 A>G | Intron14 |  | 0.093 | 0.146 | 0.65(0.40-1.05) | 0.08 |  | 0.113 | 0.146 | 0.79(0.54-1.15) | 0.22 |
|  | rs4957374 C>A | Intron14 |  | 0.211 | 0.149 | 1.60(1.07-2.39) | **0.02** |  | 0.190 | 0.149 | 1.39(0.98-1.96) | 0.07 |
|  | rs918608 T>C | Intron15 |  | 0.324 | 0.385 | 0.77(0.56-1.06) | 0.11 |  | 0.365 | 0.385 | 0.93(0.71-1.20) | 0.56 |
|  | rs1014588 G>A | Intron17 |  | 0.377 | 0.328 | 1.22(0.90-1.66) | 0.21 |  | 0.344 | 0.328 | 1.05(0.81-1.37) | 0.70 |
|  | rs3805711 C>T | Intron17 |  | 0.377 | 0.333 | 1.19(0.87-1.62) | 0.28 |  | 0.344 | 0.333 | 1.02(0.79-1.33) | 0.87 |
|  | rs3805710 A>G | Intron17 |  | 0.049 | 0.077 | 0.65(0.32-1.30) | 0.22 |  | 0.052 | 0.077 | 0.68(0.39-1.19) | 0.17 |
|  | rs9200 A>G | 3'UTR |  | 0.260 | 0.245 | 1.11(0.79-1.56) | 0.56 |  | 0.242 | 0.245 | 1.01(0.75-1.35) | 0.95 |

**P* values were adjusted for age at initial diagnosis, sex, smoking status, atopy and body mass index.

AIA, aspirin-intolerant asthma; AIA-I, intermediate aspirin-intolerant asthma; ATA, aspirin-tolerant asthma; MAF, minor allele frequency; OR, odds ratio; CI, confidence interval.
