# Supplementary material for: Genome-Wide and Follow-Up Studies Identify CEP68 Gene Variants Associated with Risk of Aspirin-Intolerant Asthma
Source: PLoS One. 2010 Nov 3;5(11):e13818. doi: 10.1371/journal.pone.0013818 (PMC2972220; doi:10.1371/journal.pone.0013818)
Supplement: Table S4 — LD coefficients (D' and r2) among CEP68 polymorphisms. (0.03 MB DOC) [file pone.0013818.s004.doc]

**Table S4.** LD coefficients (D' and *r^2^*) among *CEP68* polymorphisms

|  |  | D' | | | | | | |
| --- | --- | --- | --- | --- | --- | --- | --- | --- |
|  |  | rs2302647 C>T | rs2252867 A>G | rs12611491 A>G | rs7572857 G>A | rs2723087 T>A | rs6741255 T>C | rs10496123 G>A |
| *r^2^* | rs2302647 C>T | - | 0.97 | 0.88 | 1 | 0.97 | 0.96 | 0.92 |
|  | rs2252867 A>G | 0.90 | - | 1 | 1 | 1 | 0.99 | 0.95 |
|  | rs12611491 A>G | 0.54 | 0.65 | - | 1 | 1 | 0.98 | 0.95 |
|  | rs7572857 G>A | 0.20 | 0.19 | 0.03 | - | 1 | 1 | 1 |
|  | rs2723087 T>A | 0.90 | 1 | 0.65 | 0.19 | - | 0.99 | 0.95 |
|  | rs6741255 T>C | 0.87 | 0.97 | 0.63 | 0.19 | 0.97 | - | 1 |
|  | rs10496123 G>A | 0.21 | 0.24 | 0.15 | 0.05 | 0.24 | 0.26 | - |

LD coefficients (D' and *r^2^*) between all pairs of biallelic loci are calculated.
