# Supplementary material for: Genome-Wide and Follow-Up Studies Identify CEP68 Gene Variants Associated with Risk of Aspirin-Intolerant Asthma
Source: PLoS One. 2010 Nov 3;5(11):e13818. doi: 10.1371/journal.pone.0013818 (PMC2972220; doi:10.1371/journal.pone.0013818)
Supplement: Table S5 — Relation of the SNP genotypes in CEP68 to the decline on FEV1 in AIA and in ATA. (0.05 MB DOC) [file pone.0013818.s005.doc]

**Table S5.** Relation of the SNP genotypes in *CEP68* to the decline on FEV_1_ in AIA and in ATA.

|  | **SNP ID** | **C/C** | |  | **C/R** | |  | **R/R** | |  | ***P**** |
| --- | --- | --- | --- | --- | --- | --- | --- | --- | --- | --- | --- |
|  |  | **n** | **Decline of FEV_1_ (%)** |  | **n** | **Decline of FEV_1_ (%)** |  | **n** | **Decline of FEV_1_ (%)** |  |  |
| In AIA | rs2302647 C>T | 63 | 20.68 ± 14.99 |  | 68 | 26.69 ± 16.45 |  | 29 | 28.36 ± 16.39 |  | **0.02** |
|  | rs2252867 A>G | 59 | 21.18 ± 15.57 |  | 71 | 26.25 ± 16.02 |  | 30 | 27.55 ± 16.71 |  | 0.06 |
|  | rs12611491 A>G | 80 | 23.50 ± 15.59 |  | 69 | 27.06 ± 16.88 |  | 11 | 17.57 ± 12.76 |  | 0.97 |
|  | rs7572857 G>A | 126 | 22.63 ± 15.89 |  | 29 | 31.53 ± 15.61 |  | 5 | 34.80 ± 10.76 |  | **0.003** |
|  | rs2723087 T>A | 59 | 21.18 ± 15.57 |  | 71 | 26.25 ± 16.02 |  | 30 | 27.55 ± 16.71 |  | 0.06 |
|  | rs6741255 T>C | 58 | 21.27 ± 15.69 |  | 72 | 26.11 ± 15.95 |  | 30 | 27.55 ± 16.71 |  | 0.06 |
|  | rs10496123 G>A | 75 | 27.02 ± 16.57 |  | 72 | 22.20 ± 15.51 |  | 13 | 24.25 ± 15.85 |  | 0.22 |
|  |  |  |  |  |  |  |  |  |  |  |  |
| In ATA | rs2302647 C>T | 204 | 3.08 ± 4.84 |  | 185 | 3.95 ± 4.94 |  | 40 | 4.03 ± 4.35 |  | 0.07 |
|  | rs2252867 A>G | 198 | 3.07 ± 4.69 |  | 188 | 3.87 ± 5.07 |  | 43 | 4.28 ± 4.45 |  | 0.05 |
|  | rs12611491 A>G | 243 | 3.19 ± 4.62 |  | 162 | 3.96 ± 5.20 |  | 22 | 4.60 ± 4.42 |  | 0.07 |
|  | rs7572857 G>A | 363 | 3.50 ± 4.92 |  | 66 | 3.79 ± 4.43 |  | - | - |  | 0.53 |
|  | rs2723087 T>A | 198 | 3.07 ± 4.69 |  | 188 | 3.87 ± 5.07 |  | 43 | 4.28 ± 4.45 |  | 0.05 |
|  | rs6741255 T>C | 197 | 3.09 ± 4.71 |  | 192 | 3.82 ± 5.03 |  | 40 | 4.47 ± 4.51 |  | **0.04** |
|  | rs10496123 G>A | 188 | 3.74 ± 4.71 |  | 191 | 3.47 ± 4.91 |  | 50 | 3.09 ± 5.15 |  | 0.32 |

**P* values of regression analyses represent the co-dominant model adjusted for age at initial diagnosis, sex, smoking status and atopy.

C/C, C/R and R/R indicate the homozygote of the common allele, and the heterozygote and homozygote of the rare allele, respectively.

In case of AIA, data for decline of EFV_1_ in two patients were omitted. Decline of FEV_1_ represents mean ± SE value.
