# Supplementary material for: Genome-Wide and Follow-Up Studies Identify CEP68 Gene Variants Associated with Risk of Aspirin-Intolerant Asthma
Source: PLoS One. 2010 Nov 3;5(11):e13818. doi: 10.1371/journal.pone.0013818 (PMC2972220; doi:10.1371/journal.pone.0013818)
Supplement: Table S6 — List of other genes that were associated previously with AIA. (0.05 MB DOC) [file pone.0013818.s006.doc]

**Table S6. List of other genes that were associated previously with AIA.**

| **Gene** | **SNP** | **AA Change** | **Chr** | **Location** | **MAF (AIA)** | **ATA (MAF)** | ***P* value** |
| --- | --- | --- | --- | --- | --- | --- | --- |
| *TNFSF4* | rs3861950 | . | chr1 | intron | 0.035 | 0.116 | 0.004 |
| *TNFRSF17* | rs10451099 | . | chr16 | Promoter | 0.253 | 0.158 | 0.022 |
| *TNFRSF17* | rs2017662 | . | chr16 | coding | 0.242 | 0.157 | 0.033 |
| *TNFRSF11A* | rs3826620 | . | chr18 | intron | 0.380 | 0.485 | 0.033 |
| *TNFRSF11A* | rs1805034 | A192V | chr18 | coding | 0.315 | 0.409 | 0.039 |
| *TNFSF13B* | rs196166 | . | chr13 | Intergenic | 0.273 | 0.362 | 0.046 |
| *TNFRSF13B* | rs2274892 | . | chr17 | intron | 0.290 | 0.384 | 0.047 |
|  |  |  |  |  |  |  |  |
| *TGFBR3* | rs7550034 | . | chr1 | Intergenic | 0.155 | 0.260 | 0.012 |
| *TGFA* | rs7605323 | . | chr2 | Intergenic | 0.430 | 0.301 | 0.014 |
| *TGFB2* | rs7552931 | . | chr1 | Intergenic | 0.222 | 0.321 | 0.021 |
| *TGFBR3* | rs2810904 | . | chr1 | coding | 0.120 | 0.204 | 0.023 |
|  |  |  |  |  |  |  |  |
| *HLA-DPB1* | rs3117224 | . | chr6 | Intergenic | 0.045 | 0.111 | 0.018 |
| *HLA-DPB1* | rs1042151 | M105V | chr6 | coding | 0.087 | 0.158 | 0.038 |
| *HLA-DPB1* | rs1126513 | G40V | chr6 | coding | 0.150 | 0.227 | 0.044 |
|  |  |  |  |  |  |  |  |
| *ALOX5AP* | rs4769048 | . | chr13 | Intergenic | 0.131 | 0.217 | 0.030 |
|  |  |  |  |  |  |  |  |
| *IL-10* | rs1518111 | . | chr1 | intron | 0.275 | 0.371 | 0.048 |
|  |  |  |  |  |  |  |  |
| *ACE* | rs4309 | . | chr17 | coding | 0.395 | 0.479 | 0.077 |
|  |  |  |  |  |  |  |  |
| *TBX21* | rs11079788 | . | chr17 | intron | 0.065 | 0.056 | 0.683 |
|  |  |  |  |  |  |  |  |
| *LTC4S* | rs730012 | . | chr5 | promoter | 0.140 | 0.146 | 0.866 |

**P* values from GWAS in this study. Only SNPs with significance (*P* < 0.05) are shown.

Only SNPs with the highest significance in the *ACE*, *TBX21*, and *LTC4S* genes, which were reported to be associated previously with AIA, are shown.

AA, amino acid; MAF, minor allele frequency; AIA, aspirin-intolerant asthma; ATA, aspirin-tolerant asthma; Chr, chromosome.
