# Supplementary material for: Genome-Wide and Follow-Up Studies Identify CEP68 Gene Variants Associated with Risk of Aspirin-Intolerant Asthma
Source: PLoS One. 2010 Nov 3;5(11):e13818. doi: 10.1371/journal.pone.0013818 (PMC2972220; doi:10.1371/journal.pone.0013818)
Supplement: Figure S1 — In silico annotation of nonsynonymous rs7572857G>A (Gly74Ser). Impact on protein function and conservation across species are predicted by the SNPs3D program (http://www.snps3d.org/). (A) The higher scores of entropy and Position Specific Scoring Matrix (PSSM) indicate more tolerable to diseases. (B) The 74th amino acid of CEP68 is not highly conserved among mammals. (0.17 MB DOC) [file pone.0013818.s007.doc]

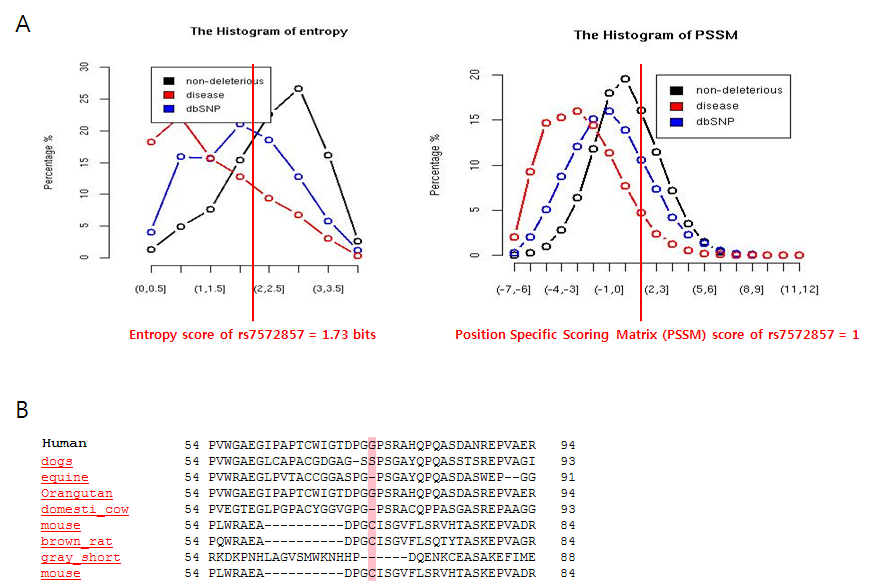


**Figure S1.** ***In silico* annotation** **of nonsynonymous rs7572857G>A (Gly74Ser).** Impact on protein function and conservation across species are predicted by the SNPs3D program (http://www.snps3d.org/). (A) The higher scores of entropy and Position Specific Scoring Matrix (PSSM) indicate more tolerable to diseases. (B) The 74^th^ amino acid of CEP68 is not highly conserved among mammals.
