# Supplementary material for: Genome-Wide and Follow-Up Studies Identify CEP68 Gene Variants Associated with Risk of Aspirin-Intolerant Asthma
Source: PLoS One. 2010 Nov 3;5(11):e13818. doi: 10.1371/journal.pone.0013818 (PMC2972220; doi:10.1371/journal.pone.0013818)
Supplement: Figure S2 — Comparison of LD from other populations for the selected CEP68 SNPs. The LDs are constructed by seven CEP68 SNPs that are equivalent with this study from HapMap (http://hapmap.ncbi.nlm.nih.gov/index.html.en). CEU, Caucasian; CHB, Chinese, JPT, Japanese; YRI, African. (0.12 MB DOC) [file pone.0013818.s008.doc]

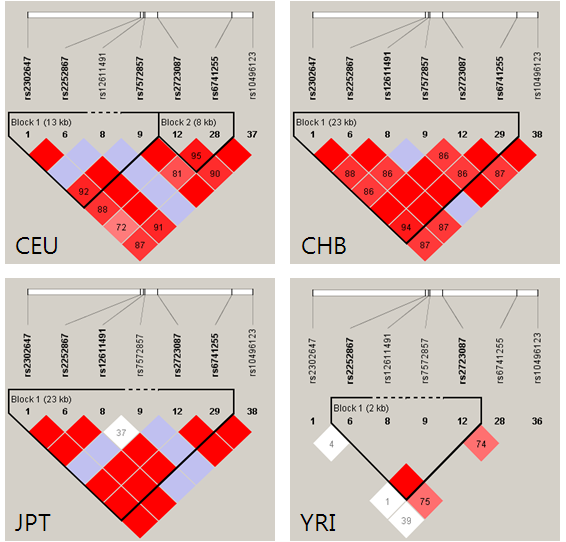


**Figure S2.** **Comparison of LD from other populations for the selected *CEP68* SNPs.** The LDs are constructed by seven *CEP68* SNPs that are equivalent with this study from HapMap (<http://hapmap.ncbi.nlm.nih.gov/index.html.en>). CEU, Caucasian; CHB, Chinese, JPT, Japanese; YRI, African.
