# Supplementary material for: Genome-Wide and Follow-Up Studies Identify CEP68 Gene Variants Associated with Risk of Aspirin-Intolerant Asthma
Source: PLoS One. 2010 Nov 3;5(11):e13818. doi: 10.1371/journal.pone.0013818 (PMC2972220; doi:10.1371/journal.pone.0013818)
Supplement: Figure S3 — Distribution of FEV1 decline rate. Distributions are calculated from the decline rate of FEV1 by aspirin provocation and its number of subjects with 5% intervals. The distribution of AIA patients with FEV1 decline rate less than 15% is due to the responders to naso-ocular or cutaneous reactions. (0.03 MB DOC) [file pone.0013818.s009.doc]

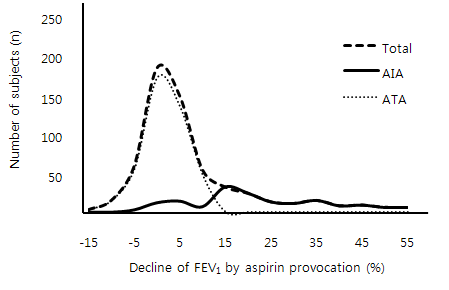


**Figure S3. Distribution of FEV_1_ decline rate.** Distributions are calculated from the decline rate of FEV_1_ by aspirin provocation and its number of subjects with 5% intervals. The distribution of AIA patients with FEV_1_ decline rate less than 15% is due to the responders to naso-ocular or cutaneous reactions.
